# Implementing supported self-management for asthma: a systematic review and suggested hierarchy of evidence of implementation studies

**DOI:** 10.1186/s12916-015-0361-0

**Published:** 2015-06-01

**Authors:** Hilary Pinnock, Eleni Epiphaniou, Gemma Pearce, Hannah Parke, Trish Greenhalgh, Aziz Sheikh, Chris J. Griffiths, Stephanie J. C. Taylor

**Affiliations:** Asthma UK Centre for Applied Research, Allergy and Respiratory Research Group, Usher Institute for Population Health Sciences and Informatics, University of Edinburgh, Doorway 3, Medical School, Teviot Place, Edinburgh, EH8 9AG UK; Centre for Primary Care and Public Health, Barts and The London School of Medicine and Dentistry, Queen Mary University of London, London, E1 2AB UK; Centre for Technology Enabled Health, Coventry University, Coventry, CV1 5FB UK; Department of Primary Care Health Sciences, New Radcliffe House, 2nd floor, Walton Street, Oxford, OX2 6GG UK; Public Health and Primary Care, Multidisciplinary Evidence Synthesis Hub (mEsh), Centre for Primary Care and Public Health, Barts and The London School of Medicine and Dentistry, Queen Mary University of London, London, E1 2AB UK

**Keywords:** Systematic review, Asthma self-management, Phase IV implementation studies, Dissemination and implementation

## Abstract

**Background:**

Asthma self-management remains poorly implemented in clinical practice despite overwhelming evidence of improved healthcare outcomes, reflected in guideline recommendations over three decades. To inform delivery in routine care, we synthesised evidence from implementation studies of self-management support interventions.

**Methods:**

We systematically searched eight electronic databases (1980 to 2012) and research registers, and performed snowball and manual searches for studies evaluating implementation of asthma self-management in routine practice. We included, and adapted systematic review methodology to reflect, a broad range of implementation study designs. We extracted data on study characteristics, process measures (for example, action plan ownership), asthma control (for example, patient reported control questionnaires, days off school/work, symptom-free days) and use of health services (for example, admissions, emergency department attendances, unscheduled consultations). We assessed quality using the validated Downs and Black checklist, and conducted a narrative synthesis informed by Kennedy’s whole systems theoretical approach (considering patient, practitioner and organisational components and the interaction between these).

**Results:**

We included 18 studies (6 randomised trials, 2 quasi-experimental studies, 8 with historical controls and 3 with retrospective comparators) from primary, secondary, community and managed care settings serving a total estimated asthma population of 800,000 people in six countries. In these studies, targeting professionals (n = 2) improved process, but had no clinically significant effect on clinical outcomes. Targeting patients (n = 6) improved some process measures, but had an inconsistent impact on clinical outcomes. Targeting the organisation (n = 3) improved process measures, but had little/no effect on clinical outcomes. Interventions that explicitly addressed patient, professional and organisational factors (n = 7) showed the most consistent improvement in both process and clinical outcomes. Authors highlighted the importance of health system commitment, skills training for professionals, patient education programmes supported by regular reviews, and on-going evaluation of implementation effectiveness.

**Conclusions:**

Our methodology offers an exemplar of reviews synthesising the heterogeneous implementation literature. Effective interventions combined active engagement of patients, with training and motivation of professionals embedded within an organisation in which self-management is valued. Healthcare managers should consider how they can promote a culture of actively supporting self-management as a normal, expected, monitored and remunerated aspect of the provision of care.

**Systematic review registration:**

PROSPERO (registration number: CRD42012002898) Accessed 24 May 2015

**Electronic supplementary material:**

The online version of this article (doi:10.1186/s12916-015-0361-0) contains supplementary material, which is available to authorized users.

## Background

Supported self-management for people with asthma, including a personal asthma action plan, reduces emergency use of healthcare resources and improves markers of asthma control [1, 2]. International guidelines prioritise establishing a patient/clinician partnership to help people take control of their asthma with guidance from healthcare professionals [3], and recommend provision of self-management education for all people with asthma [4]. Yet, nearly 25 years after guidelines first advised that ‘as far as possible patients should be trained to manage their own treatment’ [5], and despite significant promotion of self-management in healthcare policy globally [6, 7], surveys from United States, Northern Europe, and Australia estimate that less than a third of people with asthma have a personal action plan [8–12]. In 2014 the UK National Review of Asthma Deaths highlighted that half the people who died had not accessed medical help and emphasised the vital importance of asthma self-management to facilitate recognition of, and timely response to, deteriorating asthma control [13].

Most evidence supporting complex interventions, such as self-management education, is derived from randomised controlled trials (RCTs); studies of implementation in routine practice are relatively uncommon [14, 15]. Recently, however, the policy focus has shifted to the translational gap between research and practice [16], echoed by the development of a Dissemination and Implementation (D and I) research paradigm [17, 18], and a growing emphasis on ‘research impact’ [19]. These initiatives may provide the impetus required to move health service research from (often ineffective) dissemination to active translation of efficacious interventions into practical approaches for effective implementation within diverse healthcare systems [15, 20].

A range of methodologies inform the study of real-world implementation, but the crucial distinguishing feature is that the intervention is delivered by practitioners within the context of routine clinical care and accessible to all patients clinically eligible for the service (as opposed to participants selectively recruited into a research study) [14]. Outcomes should reflect this, potentially using routinely collected data to assess impact on the whole population; turnover within that population is a clinical reality [15]. Uptake and attrition in a real-world setting are important outcome measures.

**Fig. 1:**
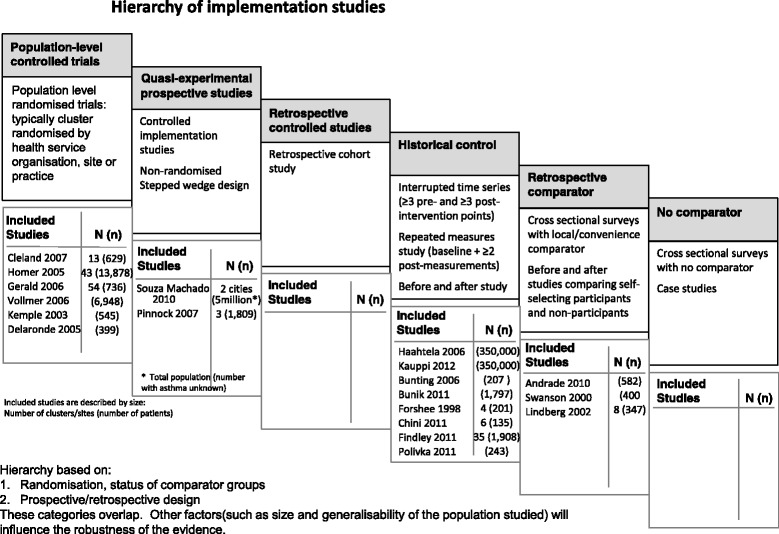
Hierarchy of implementation studies. Hierarchy based on: 1. Randomisation, status of comparator groups. 2. Prospective/retrospective design. These categories overlap. Other factors (such as size and generalisability of the population studied) will influence the robustness of the evidence

RCTs are the gold standard for establishing effectiveness, though in implementation research such trials typically randomise clusters (for example, hospitals, primary care practices, healthcare organisations) to implementing the intervention or continuing standard care. Other study designs, including quasi-experimental (for example, stepped wedge, controlled implementation studies) or studies with retrospective controls may be more practicable and affordable than large cluster RCTs, but are more open to bias [21]. Weaker designs include before-and-after studies, and uncontrolled cross-sectional studies, although these can contribute to understanding the challenges of implementing complex interventions. Qualitative and mixed-method case studies can draw out rich explanations of how and why events unfolded in a particular setting although they are a weak design for evaluating effectiveness. This hierarchy is illustrated in Fig. 1, although importantly the categories overlap, as factors such as the size and generalisability of the population studied, and reliability of routine data may influence the robustness of the findings.

This paper describes a systematic review of studies reporting the delivery of self-management interventions in populations with asthma within routine clinical care. It formed part of a larger programme of work (PRISMS) that aimed to synthesise the evidence for self-management support interventions for people with long-term conditions to inform commissioners and providers of healthcare services [22].

## Methods

The study protocol was registered with PROSPERO (registration number: CRD42012002898). The review was undertaken during 2012 to 2013 with database searches completed in August 2012 and other searches in November 2012. We followed the approach described in the Cochrane Handbook for Systematic Reviews of Interventions [23].

### Search strategy

Table 1 outlines the search strategy, the search process, and inclusion and exclusion criteria; full details are given in Additional files 1 and 2. Our initial searches in eight electronic databases, two trial registries, snowball and manual searches of key journals covered the 14 long-term conditions studied in our over-arching PRISMS study, which specifically included asthma [22]. Our basic search strategy was: ‘self-management support’ AND ‘exemplar long term conditions (specifically including asthma)’ AND ‘implementation design terms’.

**Table 1:** Search strategy and sources for the implementation review

Component	Description, inclusion/exclusion criteria, process
Population	Studies were included if self-management support was delivered to populations with one or more of the exemplar long-term conditions (asthma, chronic kidney disease, chronic obstructive pulmonary disease, dementia, depression, diabetes (Type 1 and 2), epilepsy, hypertension, inflammatory arthritis, irritable bowel syndrome, stroke, low back pain, progressive neurological disease) selected for study in our overview of the literature [22].
Intervention	We included any implementation intervention which focused on, or incorporated, strategies to support self-management, and which were delivered as part of routine clinical service. Self-management support search terms included ‘confidence’, ‘self-efficacy’, ‘responsib*’, ‘autonom*’, ‘educat*’, ‘knowledge’, ‘(peer or patient) ADJ1 (support or group)’ and ‘(lifestyle or occupational) ADJ1 (intervention* or modification* or therapy)’ as well as relevant MeSH terms.
Comparator	Typically ‘usual care’, although definition of ‘usual care’ varied between trials. The nature of the control service was noted and accommodated within our analysis.
Outcomes	Use of healthcare services (including unscheduled use of healthcare services and hospital admission rates), health outcomes (including symptom control, biological markers of disease), and process outcomes (ownership of action plans, attendance at education sessions) and intermediary outcomes (self-efficacy).
Settings	Any healthcare setting: hospital (in-patient or out-patient), community or remote (for example, web based) settings.
Study design	Implementation studies [14, 15], including a range of methodologies: population level randomised controlled trials, quasi-experimental prospective studies, retrospective controlled studies, interrupted time series, controlled before and after studies, case–control, uncontrolled before and after studies, and observational studies.
Databases	MEDLINE (1980 onwards), EMBASE (1974 onwards), CINAHL (1982 onwards), PsychINFO, AMED (1985 onwards), BNI, Database of Abstracts of Reviews of Effects and ISI Proceedings (Web of Science).
Manual searching	Patient Education and Counseling, Health Education and Behaviour and Health Education Research.
Forward citations	A forward citation search was performed on all included papers using ISI Proceedings (Web of Science). The bibliographies of all eligible studies were scrutinised to identify additional possible studies.
Unpublished and in progress studies	UK Clinical Research Network Study Portfolio (www.clinicaltrials.gov) and the Meta Register of Controlled Trials (www.controlled-trials.com)
Other exclusion criteria	We excluded papers not published in English.

### Defining and identifying implementation studies

There is no consensus on standards for defining and describing implementation studies [15, 24]. We therefore discussed and agreed key criteria for identifying relevant studies (see Additional file 2). In summary, we only included studies in which the intervention was implemented in routine clinical practice. From a practical perspective this meant the studies had to describe the introduction of an evidence-based and/or guideline recommended intervention, define eligibility for, and recruit patients to the new service (rather than recruiting eligible patients into a research study where receipt of the new service was dependent on being in the study), report uptake and attrition, be delivered by service personnel (although they could be trained specifically to deliver the intervention), and include outcomes from whole populations. We anticipated that the studies would employ a range of study designs (see Fig. 1) [14].

### Screening of titles and abstracts

Following training, one reviewer (EE, GP or HLP) reviewed titles and abstracts from the literature searches and selected possibly relevant studies with a random 10 % sample checked independently by a second reviewer (GP, HLP or HP). Disagreements were resolved by discussion or, rarely, through arbitration by a third reviewer (ST).

### Full-text screening

The full texts of all potentially eligible studies were assessed against the exclusion criteria (EE, GP or HLP) with a random 25 % sample checked independently (HP). Because of the challenges in identifying implementation studies, all papers considered relevant or where there was doubt about eligibility were discussed by the core research team (ST, HP, EE, GP, HLP), agreement reached and criteria refined.

### Assessment of methodological quality

In the absence of an instrument specifically designed for implementation studies, we used the Downs and Black checklist, which assesses methodological quality of both randomised and non-randomised studies of healthcare interventions [25]. A random 10 % sample of papers was scored independently by EE and HP; discussions to resolve disagreements on these papers improved consistency in applying the instrument, which was then applied by EE to the remaining papers. Members of the study team did not contribute to decisions to include, or the assessment of, papers on which they were authors.

### Outcomes

Reflecting international guidance on assessing asthma control [26], our primary outcomes were validated patient-reported measures of current asthma control [27] and acute exacerbations (steroid courses and/or unscheduled healthcare). We also included process measures (for example, presence of personal action plans), and intermediate measures (for example, self efficacy) relevant to provision of self-management.

### Extraction of data

One reviewer (EE) extracted data on: study design; group allocation (if applicable); setting; mode of delivery (group, individual, professional, lay-led, face-to-face, telehealthcare); recipient of the intervention (healthcare professionals, patients, parents or carers); components (education, action plans, behaviour change techniques; (tele) monitoring; written/electronic information); duration and intensity of components; follow-up; service arrangements; and clinical effectiveness and process outcomes. A second reviewer (HP) independently checked all data extracted for integrity and accuracy.

### Data analysis

We compiled a descriptive summary of the studies and, for each, the evidence for the effectiveness of implementing self-management support. Substantial heterogeneity of populations, interventions and outcomes precluded meta-analysis. We used narrative synthesis, using Kennedy *et al*’s ‘whole systems’ approach (considering patient, practitioner and service organisation components and the interaction among these) as an organising framework [28]. Classification of papers was determined by the authors’ description of the focus of their intervention.

We used harvest plots to assist the process of synthesis and to provide a visual representation of our findings [29]. For each study, a decision was made about whether the outcomes (classified as process/intermediate, asthma control, unscheduled healthcare) showed overall positive benefit, no effect, or negative effect. If several measures within one classification (for example asthma control questionnaire, days off school and use of rescue medication) had different outcomes (typically because some, but not all, outcomes showed a significant effect) a decision was taken about the over-arching outcome and the bar on the plot hatched to indicate inconsistent findings. In making this assessment, precedence was given to defined primary outcomes for which the study was adequately powered, outcomes which determined impact on the whole eligible population, and outcomes which used validated instruments and achieved clinically important differences.

### Interpretation and end-of-project workshop

The multidisciplinary core research team met regularly (usually weekly) to discuss emerging findings, and monthly steering group meetings provided further opportunities to discuss interpretation and ensure balanced conclusions. In addition, we presented our preliminary findings and draft conclusions at a multidisciplinary end-of-project workshop attended by 32 policymakers, commissioners, health service managers, healthcare professionals, academics, and patient representatives.

## Results

**Fig. 2:**
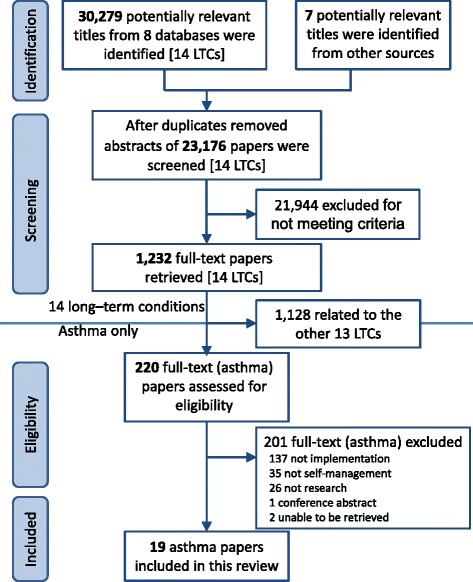
PRISMA flowchart. Note: The initial searches were combined for all the 14 LTCs in the PRISMS overview [22]. The figures for asthma are provided from the point of full text screening. LTCs, long term conditions

The papers identified (for the combined search of 14 long term conditions including asthma), the screening process and the final number of studies included, are detailed in the PRISMA flowchart (Fig. 2). On title and abstract screening, the 10 % reliability check showed 93 % agreement between reviewers. Of the 1,225 papers selected for full-text screening, 220 related to asthma of which 201 were excluded as not meeting our inclusion criteria.

### Description of the studies

Our final dataset comprised 19 papers reporting 18 studies, nine of which were from the US [30–38], four from the UK [39–42], two from Brazil [43, 44], one (two reports) from Finland [45, 46], and two from other European countries [47, 48]. Nine were conducted in primary care or community settings [30, 38–44, 48], four in managed care organisations (MCOs) [31–33, 35], one in secondary care [36], three in schools [34, 37, 47], and one was a national initiative spanning all healthcare settings [45, 46].

### Study quality and weight of evidence

Study designs varied (see Fig. 1), with six RCTs (three cluster RCTs [30, 34, 40], one preference trial [32], and two individually randomised RCTs [35, 40]), two quasi-experimental designs [41, 44], eight with historical controls (two interrupted time series [45, 46]), three repeated measures studies [31, 33, 36], and three before-and-after studies [37, 38, 47]), and three with retrospective comparators [42, 43, 48].

The size of the studies varied widely. The largest studies were a national initiative in Finland involving an estimated 350,000 people with asthma [45, 46] and a comparison between two Brazilian cities each with a population of about 2.5 million [44]. Five other studies included asthma populations in excess of 1,000 people [30, 35–37, 41]. Only three included fewer than 250 people with asthma [31, 38, 47].

Quality scores using the Downs and Black checklist (‘D and B’) ranged from 10 to 24 out of a possible 28 [25]. It became apparent that, despite its broad remit, the score prioritised several items of questionable importance in the context of implementation studies. For example, ‘representativeness of subjects invited to participate’ is not applicable if all patients are offered a clinical service (as opposed to being recruited to research). Turnover (both gains and losses in the population eligible for the clinical service) is more relevant than ‘loss to research follow up’: uptake and attrition from the clinical service are important outcomes. Questions about ‘blinding’ are irrelevant and possibly even misleading in the context of a 10-year national quality improvement programme [45, 46], in which publicity was a key part of the intervention.

In order to reflect the relative weight that should be given to the findings of individual studies, we have summarised these three attributes (design, population size, quality score) when we present data from the different studies. In addition we describe the studies in the text and tables in order of our assessment of design rigour (see Fig. 1).

### Overview of results

We classified studies according to whether the intervention as described in the paper was primarily targeted at patients, professionals, the organisation, or explicitly described components targeted at all three.

- Primarily patient education, with or without an attempt at organisational change(s) (six studies [31–35, 47]), improved some process measures, but had inconsistent impact on clinical outcomes.
- Primarily professional training, with or without an attempt at organisational change(s), (two studies [30, 39]) improved process, but had no clinically significant impact on clinical outcomes.
- Primarily organisational change (three studies [40, 41, 48]) improved process, but had little or no effect on clinical outcomes.
- A whole systems approach with components explicitly operating at patient, professional and organisational level (seven studies reported in eight papers [36–38, 42–46]) showed the most consistent improvement in both process measures and clinical outcomes.

**Fig. 3:**
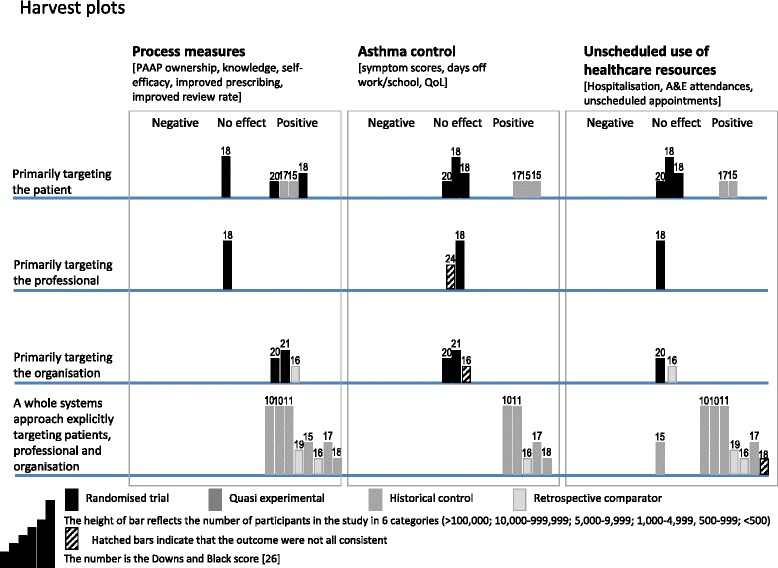
Harvest plots [29] illustrating the effectiveness on process and clinical outcomes of self-management implementation. Notes: Each bar represents a study categorised as primarily targeting patients, professionals or the organisation, or a whole systems approach explicitly targeting all three. The colour, height and number indicate the three criteria for assessing weight of evidence of individual trials. The colour of the bar indicates the study design with the more robust methodologies in darker colours. The height of the line illustrates the number of patients included in the study and the number is the Downs and Black quality score. The decisions which underpin this plot are detailed in Additional file 5

Table 2 summarises the key findings from each of the studies and Table 3 lists the authors’ reflections and lessons learned. Detailed descriptions of the interventions and findings are presented in Additional files 3 and 4. The text below summarises our synthesis, which is tabulated in Additional file 5 and illustrated in Fig. 3.

**Table 2:** Overview of the findings of the included studies

Study	Design, size and quality	Intervention			Outcomes		
		Patient	Professional	Organisation	Health service utilisation	Disease control and QoL	Process
Primarily professional training							
Cleland 2007 [39] UK Primary care	Cluster RCT. FU: 6m 13 practices: 629 adults with poorly controlled asthma, Quality score = 24	None	Intervention: one 3-hour interactive seminar vs. control	None	Not assessed	*Routine data:* SABA use and steroid courses: NS *Sub-group*: QoL (miniAQLQ): I: 6.49 (95%CI 6.40 to 6.59) vs C: 6.33 (95%CI 6.23 to 6.44) *P* = 0.03 (less than MCID of 0.5) Asthma control: NS	Not assessed
Homer 2005 [30] US Primary care	Cluster RCT. FU 12m 43 practices: 13,878 children with asthma Quality score = 18	None	Three one-day group training + two additional sessions + biweekly conference calls	Intended implementation of CCM	Admissions and ED visits: no between group differences reported	Asthma attacks and exercise limitation*:* no between group differences reported	Ownership of PAAP: I: 54% vs C: 41% (but large baseline difference) Use of preventer medication: I: 38% vs C: 39% Use of ICS I: 15% vs C: 17%
Primarily patient education							
Delaronde 2005 [32] US Managed Care Organisation	Preference RCT. FU 12 (‘opt-in’ ‘opt-out’ ‘probably’ group were randomised) 399 adults, Quality score = 20	Six-minute nurse-led telephonic case management vs usual care	None	None	Physician office visits, emergency department visits, hospitalisations: NS	*Sub-group:* No significant difference in the change in QoL (I: 0.26 vs C: 0.12) and within group changes < the MCID	Ratio of preventer to reliever medication. Increase in intervention group (0.18) was greater than in the control group (0.09) *P* = 0.04. Increase in the ‘opt-in’ group was greater at 0.29 (*P* = 0.01)
Vollmer 2006 [35] US Managed Care Organisation	RCT, 6,948 adults, (192 had live calls) Quality score = 18	Three 10-minute automated calls providing asthma review and personalised feedback	None	Provided as a service by the MCO	No between group difference in admissions/ED visits (% patients I: 4.1% vs C: 4.0% *P* = 0.88) or other unscheduled care	Asthma control: No difference in QoL (miniAQLQ I: 5.2 (SD 1.2) vs C: 5.1 (SD 1.2) *P* = 0.48) or any measure of asthma control	Medication use: No difference in ICS (% using ≥6 canisters/year I: 30.4% vs C: 29.8% *P* = 0.60)
Bunting 2006 [31] US Managed Care Organisation	Repeated measures study, eight years of routine data 207 adults, Quality score=17	One-to-one education + PAAP by a hospital based asthma educator. Sessions lasted 60 to 90 minutes + regular follow-up for five years by pharmacists.	None	Pharmacist and medication costs reimbursed by health plans.	*From insurance claims:* ED visits or hospitalisations /100 patients/y were lower during the programme (5.4, 2.6, 1.9, 5.4, 0) than in three years before (21.3, 22.2, 22.3)	Compared to baseline, at most recent follow up reduced:	PAAP ownership increased from 63% at baseline to 99% at follow-up (*P* <.0001)
						• % severe /moderate asthma B: 77% vs FU: 49% *P* <0.001	
						• working days lost B: 2.5/patient/year vs FU 0.5/patient/year	
Forshee 1998 [33] US Managed Care Organisation	Before and after study over 24 weeks 201 adults/children with poorly controlled asthma, Quality score = 15	Tailored individualised education + videos + handouts	Nurse champions were educated about asthma	None	Compared to baseline, at follow up patients had:	Compared to baseline, at follow up patients had:	Monthly reviews, knowledge and confidence (non-validated questionnaire) increased significantly for both adults and children
					• Fewer episodes of unscheduled care (*P* ≤0.01)	• Improved severity classification (*P* <0.001)	
						• Improved QoL (*P* ≤0.001)	
						• Fewer days off work B: 6.5 vs FU: 3.9 (*P* <0.05)	
Gerald 2006 [34] Inner city elementary schools	Cluster RCT, 54 schools, 736 children, Quality score = 18	6 × 30 minute group education sessions for pupils with asthma + a clinical assessment with a paediatric allergist who developed a PAAP	None	Asthma education was provided for school staff A 30 minute classroom lesson was given to all children in grades I to IV in the school	Compared to control, intervention children had no difference in:	Compared to control, intervention children had:	Compared to control, school education resulted in a statistically significant increase in knowledge (*P* <0.0001) in 17 of the 18 schools
					• ED visits/child I: 0.09 (SD 0.28) vs C: 0.10 (SD 0.31)	• No difference in absenteeism : 3.88 days/child/year (SD 3.5) vs C: 3.21 (SD 3.2).	
					• Admissions/child		
					• d: 0.04 (SD 0.19) vs C: 0.02 (SD 0.14)		
Chini 2011 [47] Italy Primary schools	Before-and-after 2,765 children: 135 with asthma, Quality score = 15	Clinical assessment and were given a PAAP with FU review at end of the year. Age-appropriate groups taught cognitive and breathing techniques	None	Lessons aimed at teachers, school personnel, parents, and schoolchildren to improve their knowledge of asthma	Not assessed	At the end of the year improved:	Not assessed
						• PedsQL: B: 2.2 (SD 0.79) vs FU: 3.5 (SD 0.73) *P* <0.001	
						• Parents’ perception of child’s QOL B: 3.1 (SD 0.6) vs FU: 3.5 (SD 0.4) *P* = 0.004	
						• Asthma symptoms (*P* <0.001)	
Primarily organisational change							
Kemple 2003 [40] UK Primary care	RCT, 545 adults, Quality score = 20	None	None	Organisational intervention enclosing PAAPs (blank=I (AAP) or personalised= I (PAAP)) with invitations to review	There were no significant differences in admissions or out-of-hours consultations over the subsequent 12 months	There were no significant differences in prescriptions of short-acting beta_2_ agonists, peak flow, steroid courses	Compared to control OR of a review (95%CI): I (AAP): OR 1.92 (1.18 to 3.11); I (PAAP): OR 2.33 (1.37 to 3.93)
						*Sub-group:* Compared to control, OR of changing RCP3Qs score: I (AAP): OR 1.43 (0.80 to 2.56); I (PAAP): OR 1.46 (0.81 to 2.61)	*Sub-group:* Compared to control OR of understanding of self-management (95%CI): I (AAP): OR 1.28 (0.66 to 2.45); I (PAAP): OR 2.20 (1.13 to 4.30)
Pinnock 2007 [41] UK Primary care	Controlled implementation trial, 1,809 adults and children, Quality score = 21	Usual asthma review, including provision (or review) of self-management (with PAAP).	Existing practice asthma nurses who already had an accredited diploma on asthma care	Three reminders to patients due a review, with an option to book a telephone or face-to-face review. Opportunistic telephone calls to non-responders.	Not assessed	*Sub-group:* Compared to the control group, patients in the TC-option group had	More patients reviewed (I: 66.4% vs C: 53.8% risk difference 12.6% (95% CI 7.2 to 17.9))
						• no difference in asthma control (ACQ mean (SD): I: 1.20 (1.00) vs C: 1.33 (1.13) mean diff 0.12 (−0.06 to 0.31)	*Sub-group:* Patients in the TC option group had greater:
							• enablement: *P* = 0.03
						• no difference in asthma QoL	• confidence managing asthma (*P* = 0.007).
Lindberg 2002 [48] Sweden Primary care	Cross-sectional survey, 8 practices: 347 adults + random sample of 20/practice for survey Quality score = 16	The ANP provided regular review, including patient asthma education including a PAAP.	The Asthma Nurse Practitioner (ANP) had specialist asthma training.	With the exception of emergency visits and the yearly follow-up visit to their physician all visits were made to the asthma nurse	Patients from ANP centre had:	*Survey (non-validated)* Patients from ANP centre were less likely to	*Clinical records*
					• No difference in hospitalisations (I: 2.2% vs C: 3.7% NS)	• wake at night (*P* <0.01)	ANP centre was:
					• Lower proportion of consultations (I: 43% vs C: 56% *P* <0.05)	• have activity limitation (*P* < 0.05)	• More likely record PF
					• 18% lower total healthcare costs.	• have ≥2 asthma attacks in 6m (*P* <0.05)	• Discuss smoking
						ANP centre patients had:	*Survey (non-validated)*
						• No difference in health status (EQ5D)	ANP centre patients were more likely to:
						• Increased sick leave.	• own PAAP (*P* <0.001)
							• use a PF meter
							• have knowledge about asthma (*P* <0.001)
A whole systems approach							
Haahtela 2006 [45] Finland Primary, secondary and community settings	10 year ITS, Population of Finland, Quality score = 10 (Note: many of the criteria did not apply)	Patient organisations arranged direct patient counselling and distributing information and resources free of charge	Education was provided for 5,300 respiratory specialists, 3,700 primary/secondary care professionals, 25,500 other healthcare professionals, 695 pharmacists	The Finnish Ministry of Social Affairs and Health recognised asthma as an important public health issue and set up the national programme	Over the 10 year programme:	Over the 10 year programme:	Over the 10 year programme:
					• Admissions fell from 110,000 to 51,000/year	• Sick leave decreased (from 2966 to 1920)	• Diagnosed asthmatics increased (from 225,000 to 350,000)
					• Deaths fell from 123 to 85/year	• Number of people with asthma receiving disability payments decreased from 7212 to 1741	• Proportion using ICS increased (33% to 85%)
					• ED visits fell	• Deaths fell from 123/year to 85/year	• Smoking levels remained constant,
					• Costs fell (from €1611 to €1031 per patient)		
Kauppi 2012 [46]	This publication reports follow on data from the Haahtela Finnish study (see previous entry). All the descriptive information is therefore the same.				In the six years after the end of the programme		In the three years after the end of the programme
					• Admissions have continued to fall (from 32,000 hospital days 15,000 hospital days)		• Prevalence of asthma has continued to rise (from 6.8% to 9.4%)
Souza-Machado 2010 [44] Brazil Community	Controlled implementation study over nine years, Population of Salvador and Recife (control city), Quality score = 11 (Note: many of the criteria did not apply)	Patient training: individual asthma education + monthly group sessions discussing asthma prevention and treatment	512 primary healthcare physicians, nurses, pharmacists, social workers and managers were trained on asthma and rhinitis	Healthcare community project. Centres offered specialist care and free medication to patients with severe asthma	At nine years:	Over the nine years: in-hospital mortality decreased from 23 deaths in 2003 to one in 2006. (In Recife the in-hospital mortality rate increased from five deaths in 2003 to 6 in 2006)	From 2003 to 2006, the programme dispensed 220,889 units of inhaled medication for asthma control. There was a strong inverse correlation between hospitalisation rates and drug dispensation
					• Hospitalisation rates per 10,000 inhabitants at nine years: Salvador: 2.25 vs Recife 17.06		
					• The decline (2003 to 2006) was greater in Salvador (−74.2%) than Recife (−22.2%) *P*<0.001		
Andrade 2010 [43] Brazil Primary healthcare network	Before and after study, 582 children (470 cases and 112 controls) Quality score = 19	Individual and group educational activities, including PAAP	Patient education provided by pharmacists and health workers but no details of their training.	Healthcare community project. Free medication	At 12 months 5% of cases compared to 34% of controls had unscheduled asthma consultations *P* <0.01.	Not assessed	The use of ICS was greater in cases (67%) than controls (not given). All cases (users of the service) had a PAAP
Bunik 2011 [38] US Secondary care paediatric clinics	Five year repeated measures study, 1,797 children clinic attendees, Quality score = 15	Asthma educators provided education about medications and provided PAAPs. Telephone FU two weeks after unscheduled care	Monthly education sessions for junior medical staff and nurses. Computer and paper prompts to facilitate structured review with PAAPs	Pre-consultation questionnaires for families, templates for asthma reviews, respiratory therapist support for providing education and PAAPs.	There was no significant change in the proportion of children with ED visits (B:6% vs FU:6%) and hospitalisations (B:3% vs FU:3%) from 2006 to 2009.	Not assessed	Children seen three years after the intervention were more likely to:
							• Be given a PAAP (aRR 2.86 (95% CI 2.60–3.20)
							• Have an assessment of severity (aRR 1.47 (95% 1.41 to 1.54)
							• Be prescribed ICS (aRR 1.11 (95% CI 1.05 to 1.19)
Swanson 2000 [42] Scotland Primary Care	Retrospective comparator study, 400 adults and children, Quality score = 16	Asthma self-management education in asthma clinic	Professional training in implementing the BTS asthma guideline	Provision of paper-based templates	Compared to baseline, at follow-up patients in intervention practices were less likely to have had an ED attendance (p<0.05) or unscheduled consultation (p<0.05)	Compared to patients in control practices, attendees at intervention practice clinics reported greater improvements in asthma symptoms (p<0.001)	Compared to control practices, at FU patients in intervention practices were more likely to:
							• have and follow a PAAP (*P* <0.01)
							• have attended a review (*P* <0.05)
Findley 2011 [37] US Community day care centres for pre-school children	Before-and-after study 35 centres, 1,908 children and their families, Quality score = 17	Parents received asthma education from parent mentors and a PAAP, and were encouraged to talk with their child’s physician. Children played activities and games on asthma triggers	Professionals of children enrolled in the programme were offered. Physician Asthma Care Education (PACE) training	The centre staff received training on asthma and asthma management (including creating an ‘asthma-friendly centre’), identifying children with asthma, arranging a PAAP and handling emergencies	At 9 to 12 months the proportion of children with:	At 9 to 12 months the proportion of children with:	At 9-12 months:
					• Hospitalisations fell from 24% to 11% (*P* <0.001)	• Day-care absences reduced (56% to 38%)	• PAAP use increased from 47% to 70%
					No ED visits increased from 25% to 53% (*P* <0.001).	• No night-symptoms increased (19% to 52%) (*P* <0.001)	• Staff knowledge increased 49% to 82%
						• No day symptoms: increased ( 22% to 59%) (*P* <0.001)	• Parents’ knowledge increased 62 to 79%;
							• Parents’ confidence increased from 57% to 81% (*P* <0.001);
Polivka 2011 [38] US Deprived community	Before-and-and after study, 243 children and their families, Quality score = 18	Environmental assessment home repairs, educational home visits to reduce asthma triggers, and provide asthma education and PAAPs	Professionals completed the National Center for Healthy Homes practitioners’ course and an asthma educator course.	Costs included repair work, contractors, supplies for assessment and education provided to participants	At two years children had:	At two years children had fewer:	At two year follow up:
					• fewer emergency consultations (*P* <0.001)]	• day and night symptoms *P* <0.001	• PAAP ownership increased B: 44% vs FU: 67% *P* = 0.007
					• no difference in admissions *P* = 0.229	• days with activity limitation (*P* <0.001)]	• asthma knowledge increased (*P* <0.001)
						• mean days off school B: 5.3 (SD 9.2) vs FU: 1.4 (SD 2.7) *P* <0.001	• Caregiver
							• self-efficacy increased (*P* <0.001)

*B* baseline; *C* control group; *CCM* Chronic Care Model; *d* day; *ED* Emergency Department; *FU* follow up; *hr* hour; *I* intervention group; *ICS* inhaled corticosteroid; *LABA* long acting beta-agonist *LTC* long-term condition; *m* month; *MCID* minimum clinically important difference; *min* minute; *miniAQLQ* mini QoL questionnaire; *MCO* Managed Care Organisation; *NS* not significant; *PAAP* personalised asthma action plan; *PedsQL* Pediatric Quality of Life Inventory 4.0; *QoL* quality of Life; *RCT* randomised controlled trial; *SABA* short acting beta-agonist; *w* week; *y* year

**Table 3:** Study authors’ reflections and lessons learned

	Practical lessons from the authors’ reflections on the process of implementing complex self-management support interventions in routine clinical care.
•	Effective patient self-management education needs to be supported by regular reviews [31, 37, 44], underpinning a partnership with patients [37]. In addition to education, aligning with patients’ perceived needs [35, 40] and preferences [32, 35].
•	Only a proportion of people accept the offer of self-management education, and all studies reported an attrition rate. For many interventions, especially those delivered in deprived communities, recruiting and retaining patients was a major challenge [37, 38]. Financial incentives (free access to care, free prescriptions, favourable insurance premiums, free patient resources) were potential strategies for increasing engagement [31, 37, 38, 43–45].
•	The use of telephone interventions may overcome some of the practical barriers to participation in self-management programmes [32, 35, 36, 41].
•	Achieving change is a challenge, even in well-motivated teams [30]. There is a need to support professionals as they integrate new behaviour into practice [39]. Promising approaches include collaboratives, and plan/do/study/act (PDSA) cycles [30, 36], and introduction of self-management support as a component of improved chronic care [31, 43–46].
•	There is a need for regular oversight and frequent reviews to ensure intervention fidelity and respond to evolving situations [45]. Frequent staff turnover can be a particular challenge which needs to be addressed [30, 34], to ensure that skills are not lost.
•	Professional training in supporting self-management [36, 37, 45, 46], collaborative multidisciplinary working [36, 45], with good communication and referral systems between professionals [44], and involving existing staff members in the design and implementation of interventions [33, 36, 42] are potentially important ingredients of implementing self-management support.
•	A team approach involving the community (and schools) was seen as essential to the success of projects in deprived, minority communities [34, 37, 47].
•	A key facilitator highlighted by several authors is the commitment of the healthcare system [41, 43–46] and/or local practice or clinic [36, 41, 42], with on-going evaluation [44–46].
•	There are practical barriers if on-going funding or resources (including time) are insufficient to enable complex interventions to be sustained [30, 36, 38].
•	Technological solutions (such as computerised cognitive behaviour therapy programmes, automated telephone calls) are being explored and show some promise [35].

### Mainly focussed on patient education

Six studies described interventions directed primarily at educating patients. Four were promoted and funded by US MCOs [31–33, 35], and two were in schools (US and Italy) [34, 47].

Delaronde 2005 [32], (RCT/patient preference study, 399 participants, D and B = 20) and Vollmer 2006 [35], (RCT, 6,948 participants, D and B =18) both provided telephone-based self-management support interventions to adult members of their MCO. Neither improved clinical outcomes, although Delaronde 2005 observed a significant improvement in the ratio of preventer to reliever treatment in both intervention groups [32]. Patient motivation was important: those who had ‘opted-in’ complied with, and achieved more benefit from, the nurse-led programme than the group randomised to the intervention [32]. In contrast, compliance with the automated telephone calls used in Vollmer 2006 was very low and the intervention had no impact on any outcome [35].

In smaller studies using methodologically weaker designs, Bunting 2006 [31] (five-year repeated measures study, 207 participants, D and B = 17) and Forshee 1998 [33] (before-and-after study, 201 participants, D and B = 15), reduced unscheduled healthcare [31, 33] and demonstrated improved control [31] or quality of life [33] when self-management education was provided by asthma educators [31] or nurses [33] alongside regular clinical review.

#### School-based interventions

The two school-based interventions provided teaching for families, children and staff as well as systematic identification of children with asthma [34, 47]. Gerald 2006, (cluster RCT, 54 schools, 736 children, D and B = 18) targeted low-income African-American elementary school children, and showed no differences in school absences, or use of emergency healthcare [34]. Challenges identified by authors to explain their findings included the high turnover in staff and pupils at the inner-city schools, and limited parental involvement. Using a weaker study design, Chini 2011 (before-and-after study, 135 children, D and B = 15) demonstrated improved asthma control in primary school children in Rome, over the year of the intervention [47].

### Mainly focused on training of healthcare professionals

Two cluster RCTs described interventions directed primarily at professionals [30, 39]. Cleland 2007, (cluster RCT, 13 practices, 629 patients, D and B = 24), provided a single workshop on communication skills and advice on formulating action plans to UK primary care asthma nurses [39]. In the US, Homer 2005 (cluster RCT, 43 practices, 13,878 children, quality score = 18) trained healthcare professionals in a quality improvement intervention [30]. Neither had a clinically important effect on clinical outcomes, although intervention practices in Homer 2005 provided more action plans [30]. Challenges included limited engagement with the training and audit programme [30], and lack of organisational support to enable the trained nurses to implement self-management in practice [39].

### Mainly focused on organisational change

Three interventions promoted structured reviews with a nurse whose remit included self-management education and the provision of personal action plans [40, 41, 48]. In UK primary care, Kemple 2003, (RCT, 545 participants, D and B = 20) [40] facilitated reviews by sending blank action plans with postal reminders and Pinnock 2007 (controlled implementation trial, 1,809 participants, D and B = 21) [41] offered a telephone asthma review service. In both trials, process and/or intermediate measures improved in the intervention groups, but neither trial showed a difference in asthma control or use of healthcare resources [40, 41].

Using a weaker design from Swedish primary care, Lindberg 2002, (cross-sectional study, 347 participants, D and B = 16) demonstrated that a nurse-led asthma service was associated with provision of action plans, and fewer asthma symptoms than in seven comparator practices [48].

### Explicitly encompassing a whole systems approach

Seven studies evaluated a whole systems approach that explicitly addressed patient, professional and system level interventions [36–38, 42–46].

Four of these studies used national or regional admissions data to observe the impact of multifaceted public health programmes [42–46]. The Finnish programme (10-year interrupted time series analysis, asthma population approximately 350,000, D and B = 10) included a strong focus on self-management education in the context of nationwide professional development and system-level expectations of raising the quality of asthma management [45, 46]. National data over the decade of the intervention showed increased use of preventer medication and a reduction in asthma deaths (from 123 to 85 a year) and hospital admissions (from 110,000 to 51,000 a year) [45] which was maintained after the end of the programme [46].

Souza-Machado 2010, (controlled implementation study, city populations of approximately 2.5 million, D and B = 11) reported an initiative supported by the Brazilian Ministry of Health and city authorities, which promoted individual and/or group patient education, training for family practitioners and pharmacists, and free consultations and prescriptions [44]. The initiative was associated with a rapid reduction in asthma admissions and mortality in contrast to rising mortality and small reduction in admissions in a matched control city. Using a weaker study design, Andrade 2010, (retrospective comparator study, 582 children, D and B = 19) reported a reduction in admissions after a similar intervention in another Brazilian city [43].

Bunik 2011, (five year repeated measures study, 1,797 clinic attendees, D and B = 15) evaluated a six-month quality improvement programme in US secondary care in which a multidisciplinary team met fortnightly to promote professional training, patient education and decision support strategies [36]. Action plan ownership increased but there was no impact on unscheduled care. Swanson 2000 [42], (retrospective comparator study, 400 patients, D and B = 16) demonstrated increased ownership of action plans, and reduced hospitalisations in practices participating in a multi-faceted initiative to promote self-management in UK primary care.

Two before-and-after studies observed improved markers of asthma control and reduced unscheduled care in multi-level self-management interventions targeting deprived communities [37, 38]. In the context of pre-school centres, Findlay 2011 [37], (before and after study, 35 centres, 1,908 families, D and B = 17) recorded the greatest benefits in children exposed to the combination of pre-school centre, parent and physician components of the intervention. Polivka 2011 [38], (before and after study, 243 families, D and B = 18) provided self-management education in the context of an intervention focused on poor housing (including funding repairs).

## Discussion

### Statement of principal findings

This review has shown that complex whole systems interventions that explicitly address patient education, professional training and organisational commitment are associated with improvement in process measures [36–38, 42–47], markers of asthma control [37, 38, 42, 44, 45], and reduced use of unscheduled healthcare [37, 38, 42–46]. Large scale initiatives that include collaborations with national or regional authorities and health services can reduce hospital admissions [43–46], deaths [44, 45], and time off work [45]. Quality improvement programmes in individual practices or services can improve ownership of personal asthma action plans [36–38, 42], and reduce morbidity [37, 38, 42].

Our findings (illustrated in Fig. 3) also demonstrate that, individually, the separate components (professional, patient, organisation) of comprehensive self-management support do not appear to be sufficient consistently to improve outcomes in asthma. Improving professionals’ knowledge on its own does not improve clinical outcomes [30, 39]. Targeting the organisation to facilitate structured reviews improves process measures but does not impact on clinical outcomes [40, 42]. Targeting the patient is related to significant changes in some process and intermediate measures (increase in knowledge [34], ownership of an action plan [30], or improved ratio of preventer to reliever medication [32],) with variable effects on clinical outcomes [31, 33, 35].

### Strengths and limitations

The key strength of this study is that it focused on a relatively neglected phase in the assessment of complex interventions [14, 15]. The lack of clearly defined terminology to describe implementation research made it difficult to identify sensitive and specific search terms. We used a broad search strategy (and screened 30,279 citations), but nevertheless may have missed some implementation studies. We defined inclusion and exclusion criteria to determine whether a study was actually assessing the implementation of an intervention, but the lack of reporting guidelines in this area [15] meant that the status of a study was not always clear. We may, therefore, have rejected some relevant papers because key information was not available, although doubtful papers were not rejected without discussion amongst the core team.

Routinely collected data were used in 10 studies [31, 32, 34–36, 39–41, 44–46], and some authors commented that information from clinical records may have been incomplete or misleading [34, 37, 39, 41]. Use of routine data, however, allows the ‘real world’ effectiveness of an intervention to be assessed using data from whole clinically eligible populations. Many studies included a broad range of outcomes in each of the categories in our Harvest plot. Combining these, potentially heterogeneous, findings involved some interpretation. To minimise the subjectivity of this process, we specified criteria (that is, defined primary outcomes, data from whole populations, adequate power, validated measures and minimum clinically important difference) that enabled us to prioritise outcomes when the results conflicted. For clarity, we hatched the three columns of the Harvest plot that combined inconsistent findings (See Additional file 5: Table S5 for details). Different decisions in these three cases would not have changed our overall conclusions.

This review was part of a large commissioned, policy-focused overview, and time constraints meant that initial screening of titles and abstracts was conducted by a single reviewer. However, we undertook training at each stage, instituted systematic checks (10 % of abstracts, 25 % of full-text screening) and checked all data extraction. Similarly, for practical reasons we excluded non-English language publications.

### The challenge of reviewing implementation research

Overall, the evidence base for asthma self-management support was more extensive and of better quality than for the other 13 long term conditions (listed in Table 1) we reviewed in the PRISMS study (data not presented) [22]. However, some studies in our asthma sample were methodologically less robust and whilst they offer some useful insights, they need to be interpreted with caution. There is no definitive classification of the diverse methodologies appropriate to implementation research (merely the implication that diverse methodologies will be appropriate [14]). We, therefore, built on existing literature [49] to develop a hierarchy of evidence (Fig. 1). We also had to accommodate extreme variation in the size of the asthma populations studied (from 135 schoolchildren [47] to a national population of approximately 350,000 people [45]). The D and B quality score did not reflect many of the key quality criteria of these implementation studies. Reporting guidelines for implementation research are currently being developed [24], work that may lead to the development of a quality checklist specifically designed to assess diverse implementation studies.

We reflected all these variables in our descriptions of our findings, so that outcomes from pragmatic randomised trials [30, 32, 34, 35, 39, 40], or from national interrupted time series analyses [45, 46], or a city-wide controlled implementation study [44] were given more credence than smaller, less robust or poorer quality studies.

Heterogeneity precluded meta-analysis but use of ‘harvest plots’, developed to illustrate the ‘differential effects of population-level interventions’ and particularly relevant for informing policy-makers, commissioners and health service managers [29], allowed us to illustrate our findings giving differential weight to different designs, size and quality of study.

### Interpretation of findings

Our findings strongly support a whole systems approach to implementing supported self-management for people with asthma [28], as a key component of high quality, proactive care [50]. Less effective interventions were characterised by the targeting of only one component of the system (professionals or patients or organisations) and sometimes by limited intensity (too little, for too short a time) of the intervention. Effective interventions tended to be multi-faceted and multi-disciplinary; actively engaging patients, and training and motivating professionals within the context of an organisation that prioritised, actively supported and monitored self-management. Future research should focus on how such whole systems interventions may be integrated into the routine care of people with asthma (including adults, children, people with additional co-morbidities and demographically or culturally diverse communities), and evaluate the clinical and cost-effectiveness of this approach.

Authors of the included studies identified a number of factors associated with the successful implementation of self-management support (see Table 3): commitment of the local healthcare system [43–45] and/or the local practice or clinic [42] to this model of care; professional training in self-management [36, 37, 45]; on-going evaluation and audit [45]; collaborative multidisciplinary working [36, 44, 45]; effective patient education supported by regular reviews [37, 44]; and partnership with patients [37]. Our findings suggest that the culture of the organisation is pivotal, as it underpins and enables integration of self-management principles into the routines of clinical care, thereby allowing the impact of patient and professional interventions to be realised [44–46]. This resonates with Greenhalgh *et al*’s finding that individual practitioners can adopt an organisational-level innovation only if the organisational structure, culture, climate and resources are conducive to such adoption [51].

### The context of high quality asthma care

Many of the interventions in the included studies were introduced in the context of generally improving services for people with asthma. Specific examples are the national programme in Finland [45, 46], the regional programmes in Brazilian cities [44] and a local initiative in Scotland [42]. In each of these, self-management was highlighted as a core component by the authors [42, 44, 45], but other aspects of the service improvement will have contributed to the improved outcomes. This reinforces our key conclusion: supported self-management is most likely to be effective when it is implemented as a core component of proactive routine care which trains and empowers professionals to deliver and patients to embrace self-management.

## Conclusions

The RCT evidence for the efficacy of self-management in asthma is extensive and overwhelmingly positive [1–4, 22]. Our findings suggest that self-management support can be implemented effectively in routine practice with significant improvements in morbidity and even mortality. Effective initiatives are promoted by policies that ensure meaningful adoption by health services, provide professional training and support, and focus on self-management education for patients in the context of high quality proactive disease management. A parallel synthesis of heath economic evidence concluded that in respiratory disease there was a consistent reduction in healthcare utilisation [52], which may mitigate the cost of delivering multi-level self-management support interventions. Commissioners and providers of services for people with asthma should consider how they can promote a culture of actively supporting self-management as a normal, expected, monitored and rewarded aspect of the provision of care.

## Additional files

Additional file 1:
**Search Strategy.** 1.1 Basic search strategy for all databases. 1.2 Detailed search terms: specific LTCs. 1.3 Search terms: specific databases. 1.4 Self-management support terms. Additional file 2:
**Exclusion criteria.**
Additional file 3:
**Description of studies, participants and service implementation.**
Additional file 4:
**Summary of the findings of the included studies.**
Additional file 5:
**All results as reported in the included papers and the decision process underpinning the Harvest plot.**

## Abbreviations

D and B: Downs and Black checklist
D and I: dissemination and implementation research
MCO: managed care organisation
RCT: randomised controlled trial
UK: United Kingdom
US: United States
