# Supplementary material for: Implementing supported self-management for asthma: a systematic review and suggested hierarchy of evidence of implementation studies
Source: BMC Med. 2015 Jun 1;13:127. doi: 10.1186/s12916-015-0361-0 (PMC4465463; doi:10.1186/s12916-015-0361-0)
Supplement: Additional file 1: — Search Strategy. 1.1 Basic search strategy for all databases. 1.2 Detailed search terms: specific LTCs. 1.3 Search terms: specific databases. 1.4 Self-management support terms. [file 12916_2015_361_MOESM1_ESM.docx]

Additional file 1. Search Strategy

1.1 Basic search strategy for all databases

Generic LTC search terms **OR** specific exemplar LTC keywords

**AND** Phase IV implementation studies search terms **OR** MESH terms for each database

**AND** generic self-management search terms **OR** self-management components

**NOT** guidelines **NOT** drug **NOT** Phase 3 or Phase III

All searches in [Title/Abstract]

| **Specific phase IV studies search term** | |
| --- | --- |
|  | ((implement* or effectiveness or clinical or ‘Phase IV’ or ‘Phase 4’ or efficacy or real-world or ‘real world’ or ‘field effectiveness’ or field-effectiveness or population or pragmatic or evidence-based or ‘evidence based’ or evaluation* or real-life or ‘real life’) adj1 (trial* or stud* or research or intervention*)) |
|  | ((case-control or quasi-experimental  or ‘quasi experimental’ or ‘interrupted time series’ or ‘uncontrolled before and after’ or ‘controlled before and after’ or before-and-after or ‘before and after’ or cluster-randomi* or ‘cluster randomi*’) adj1 (trial* or stud* or research or intervention*)) |
|  | (Real-world or ‘real world’ or routine or normal or nationwide) adj1 (setting* or practice or context*) |
|  | “Routine clinical care” |
|  | “quality improvement” |

| **MESH terms for each database** | |
| --- | --- |
| Medline/Pubmed | - Clinical trials, phase IV as topic/ - Evidence-Based Practice/ - Evidence-Based Medicine/ - Evidence-Based Nursing/ - Comparative effectiveness research/ - Disease management/ - Patient education as topic/ |
| AMED | - exp Patient education/ - exp Quality of health care/ - Chronic Disease/ - Disease management/ - Evidence based medicine/ |
| BNI | None |
| CINAHL | - Prospective studies/ - Nonrandomised trials/ - Behavioral research/ - Disease management/ - Outcomes research/ - Quality of care research/ - Summative Evaluation Research/ - Evaluation research/ - exp Clinical research/ - Medical practice, evidence-based/ - Professional practice, evidence-based/ - Nursing practice, evidence-based/ |
| EMBASE | - disease management/ - treatment outcome/ - diabetes education/ - patient education/ - Outcomes research/ - Outcome assessment/ |
| PsyclNFO | - Client education/ - Evidence based practice/ - Evidence based medicine/ - Telemedicine/ - Disease management/ - Cohort analysis/ - Educational program evaluation/ |

1.2 Detailed search terms: specific LTCs

| **Asthma** | | | | | | |
| --- | --- | --- | --- | --- | --- | --- |
| **Thesaurus Terms** | **Medline** | **AMED** | **BNI** | **CINAHL** | **EMBASE** | **PsychINFO** |
|  | Asthma/ | Asthma/ | Asthma/ | Asthma/ | Asthma/ | Asthma/ |
|  | Respiratory Hypersensitivity/ |  |  |  |  |  |
|  | Bronchial Spasm/ |  |  |  |  |  |
|  | Bronchoconstriction/ |  |  |  |  |  |
|  | ((bronchial* or respiratory or airway* or lung*) ADJ3 (hypersensitive* or hyperreactiv* or allerg* or insufficiency)) | | | | | |
|  | Bronch* ADJ3 (constrict* or spas*) | | | | | |
|  | Asthma* | | | | | |
|  | Wheez* | | | | | |
|  | Bronchoconstrict* | | | | | |
|  | Antiasthma* | | | | | |
|  | Anti-asthma* | | | | | |
|  | ‘Respiratory sounds’ | | | | | |
|  | ‘Bronchial hyperreactivity’ | | | | | |
|  | Bronchospas* | | | | | |

| **Chronic Kidney Disease** | | | | | | |
| --- | --- | --- | --- | --- | --- | --- |
| **Thesaurus Terms** | **Medline** | **AMED** | **BNI** | **CINAHL** | **EMBASE** | **PsychINFO** |
|  | Kidney Failure, Chronic/ | Kidney Failure Chronic/ | Dialysis/ | Kidney failure, chronic/ | Chronic kidney disease/ | Kidney disease/ |
|  | Renal replacement therapy/ |  | Kidney Disorders/ |  | Chronic kidney failure/ |  |
|  | ((Kidney or renal) ADJ2 (disease* or failure or insufficien* or dialysis)) | | | | | |
|  | (kidney or renal) ADJ chronic | | | | | |
|  | H?mofiltration | | | | | |
|  | Dialysis | | | | | |
|  | Predialysis | | | | | |
|  | H?modialysis | | | | | |
|  | ‘Renal replacement therapy’ | | | | | |
|  | CKD or CAPD or CCPD or APD or CRF or CRD or ESKD or ESRD or ESFF or ESRF | | | | | |

| **Chronic Obstructive Pulmonary Disease** | | | | | | |
| --- | --- | --- | --- | --- | --- | --- |
| **Thesaurus Terms** | **Medline** | **AMED** | **BNI** | **CINAHL** | **EMBASE** | **PsychINFO** |
|  | Pulmonary Disease, Chronic Obstructive/ | Pulmonary Disease Chronic Obstructive/ | COPD/ | Pulmonary Disease Chronic Obstructive/ | Chronic Obstructive Lung Disease/ | Chronic Obstructive Pulmonary Disease/ |
|  | (Obstruct* ADJ3 (pulmonary or lung* or airway* or airflow* or bronch* or respirat*)) | | | | | |
|  | Chronic* ADJ3 bronchiti* | | | | | |
|  | ‘Chronic Obstructive Pulmonary Disease’ | | | | | |
|  | ‘Chronic Obstructive Airways Disease’ | | | | | |
|  | ‘Lung disease*’ | | | | | |
|  | Emphysema* | | | | | |
|  | COPD or CAL or COAD or COLD or COBD or AECB | | | | | |

| **Dementia** | | | | | | |
| --- | --- | --- | --- | --- | --- | --- |
| **Thesaurus Terms** | **Medline** | **AMED** | **BNI** | **CINAHL** | **EMBASE** | **PsychINFO** |
|  | Dementia/ | Dementia/ | Dementia/ | Dementia/ | Dementia/ | Dementia/ |
|  | Alzheimer Disease/ | Delirium/ |  |  |  |  |
|  | Delirium/ |  |  |  |  |  |
|  | Delirium, dementia, amnestic, cognitive disorders/ |  |  |  |  |  |
|  | ((Cerebr* or brain or cogniti*) ADJ2 (deteriorat* or insufficient* or disease or syndrome or impair*)) | | | | | |
|  | ‘Mild cognitive impairment’ | | | | | |
|  | ((memory* or mental*) and (declin* or deteriorat* or impair* or los*)) | | | | | |
|  | Pick* ADJ2 disease | | | | | |
|  | Lewy* ADJ2 bod* | | | | | |
|  | Dement* | | | | | |
|  | Deliri* | | | | | |
|  | Alzheimer* | | | | | |
|  | ‘Primary progressive aphasia’ | | | | | |
|  | Binswanger* | | | | | |
|  | ‘Benign senescent forgetfulness’ | | | | | |

| **Depression** | | | | | | |
| --- | --- | --- | --- | --- | --- | --- |
| **Thesaurus Terms** | **Medline** | **AMED** | **BNI** | **CINAHL** | **EMBASE** | **PsychINFO** |
|  | Depression/ | Depression/ | Depression/ | Depression/ | Depression/ | Major Depression/ |
|  | Depressive Disorder/ |  |  |  |  |  |
|  | Depress*or Dysthymi* | | | | | |
|  | ‘Major Depressive Disorder’ or MDD | | | | | |
|  | ‘Unipolar Depression’ | | | | | |
|  | (Adjustment or mood or affective) adj1 (disorder or symptoms) | | | | | |

| **Diabetes Mellitus** | | | | | | |
| --- | --- | --- | --- | --- | --- | --- |
| **Thesaurus Terms** | **Medline** | **AMED** | **BNI** | **CINAHL** | **EMBASE** | **PsychINFO** |
|  | Diabetes mellitus, type 1/ | Diabetes Mellitus/ | Diabetes/ | Diabetes mellitus, type 1/ | Diabetes Mellitus/ | Diabetes Mellitus/ |
|  | Diabetes mellitus, type 2/ |  |  | Diabetes mellitus, type 2/ |  |  |
|  | Insulin resistance/ |  |  | Diabetic patients/ |  |  |
|  | Diabetic ketoacidosis/ |  |  |  |  |  |
|  | ((diabet* or dm) ADJ5 (typ* ADJ3 (one or ‘1’ or I))) | | | | | |
|  | ((diabet* or dm) ADJ5 (typ* ADJ3 (two or ‘2’ or II))) | | | | | |
|  | (Insulin or noninsulin or non-insulin) ADJ2 (resistan* or depend*) | | | | | |
|  | Diabet* | | | | | |
|  | DM or DM1 or DM2 or T1D or T1DM or T2D or T2DM or NIDDM or IDDM or MODY | | | | | |
|  | ‘Glucose ?tolerance’ | | | | | |

| **Epilepsy** | | | | | | |
| --- | --- | --- | --- | --- | --- | --- |
| **Thesaurus Terms** | **Medline** | **AMED** | **BNI** | **CINAHL** | **EMBASE** | **PsychINFO** |
|  | Epilepsy/ | Epilepsy/ | Epilepsy/ | Epilepsy/ | Epilepsy/ | Epilepsy/ |
|  | Seizures/ | Seizures/ | Seizures/ | Seizures/ |  |  |
|  | Epilep* | | | | | |
|  | Seizure* | | | | | |
|  | Aura* | | | | | |
|  | Convulsion* | | | | | |

| **Hypertension** | | | | | | |
| --- | --- | --- | --- | --- | --- | --- |
| **Theasaurus Terms** | **Medline** | **AMED** | **BNI** | **CINAHL** | **EMBASE** | **PsychINFO** |
|  | Hypertension/ | Hypertension/ | Blood pressure/ | Hypertension/ | Hypertension/ | Hypertension/ |
|  | Hypertens* | | | | | |
|  | Blood adj1 pressure | | | | | |

| **Irritable Bowel Syndrome** | | | | | | |
| --- | --- | --- | --- | --- | --- | --- |
| **Thesaurus Terms** | **Medline** | **AMED** | **BNI** | **CINAHL** | **EMBASE** | **PsychINFO** |
|  | Irritable bowel syndrome/ | Irritable bowel syndrome/ | Irritable bowel syndrome/ | Irritable bowel syndrome/ | Irritable bowel syndrome/ | Irritable bowel syndrome/ |
|  |  |  |  |  | Irritable colon/ |  |
|  | ((Irritable or functional or spastic) adj1 (bowel or colon)) | | | | | |
|  | IBS | | | | | |
|  | ‘Mucous colit*’ | | | | | |
|  | ‘Colon* disease*’ | | | | | |
|  | ‘Gastrointestinal syndrome*’ | | | | | |
|  | ‘Functional gastrointestinal’ | | | | | |

| **Inflammatory Arthropathies and Autoimmune Conditions** | | | | | | |
| --- | --- | --- | --- | --- | --- | --- |
| **Rheumatoid Arthritis/ Psoriatic Arthritis/ Ankylosing Spondylitis/ Systemic Lupus Erythematosus** | | | | | | |
| **Thesaurus Terms** | **Medline** | **AMED** | **BNI** | **CINAHL** | **EMBASE** | **PsychINFO** |
|  | Arthritis, Rheumatoid/ | Arthritis Rheumatoid/ | Arthritis and rheumatism/ | Arthritis, Rheumatoid/ | Rheumatoid Arthritis/ | Rheumatoid Arthritis/ |
|  | Arthritis, psoriatic/ |  |  | Arthritis, psoriatic/ | Psoriatic arthritis/ | Arthritis/ |
|  | Spondylitis, Ankylosing/ | Spondylitis Ankylosing/ |  | Spondylitis, Ankylosing/ | Ankylosing Spondylitis/ |  |
|  | Lupus Erythematosus, Systemic/ | Lupus Erythematosus Systemic/ | Systemic Diseases/ | Lupus Erythematosus, Systemic/ | Systemic Lupus Erythematosus/ | Lupus/ |
|  | ((rheumatoid or rheumatoid or revmatoid or rheumatic or rheumatic or revmatic or rheumat* or reumat* or revmarthrit*) ADJ3 (arthrit* or artrit* or diseas* or condition* or nodule*)) | | | | | |
|  | RA | | | | | |
|  | ‘Still* disease’ | | | | | |
|  | (psoria* ADJ1 (arthriti* or arthropath*)) | | | | | |
|  | ‘Ankylosing Spondylitis’ | | | | | |
|  | Lupus | | | | | |
|  | SLE | | | | | |

| **Low Back Pain** | | | | | | |
| --- | --- | --- | --- | --- | --- | --- |
|  | **Medline** | **AMED** | **BNI** | **CINAHL** | **EMBASE** | **PsychINFO** |
|  | Low back pain/ | Low back pain/ | Back pain/ | Low back pain/ | Low back pain/ | Back pain/ |
|  | ‘Low* back pain*’ | | | | | |
|  | Lumbago | | | | | |
|  | ‘Low* backache*’ | | | | | |
|  | Sciatica or Lumbosacral or Dorsalgia or Spondylosis | | | | | |
|  | Lumbar ADJ2 pain | | | | | |
|  | Back ADJ2 pain | | | | | |

| **Progressive Neurological Disorders** | | | | | | |
| --- | --- | --- | --- | --- | --- | --- |
| **Motor Neurone Disease/ Multiple Sclerosis/ Parkinson’s Disease** | | | | | | |
| **Thesaurus Terms** | **Medline** | **AMED** | **BNI** | **CINAHL** | **EMBASE** | **PsychINFO** |
|  | Motor Neuron Disease/ | Motor Neuron Disease/ | Motor Neurone Disease/ | Motor Neuron Diseases/ | Motor Neuron Disease/ | Nervous System Disorders/ |
|  | Multiple Sclerosis/ | Multiple Sclerosis/ | Multiple Sclerosis/ | Multiple Sclerosis/ | Multiple Sclerosis/ | Motor Neurons/ |
|  | Parkinson Disease/ | Parkinson Disease/ | Parkinson Disease/ | Parkinson Disease/ | Parkinson Disease/ | Neurodegenerative Diseases/ |
|  |  |  |  |  |  | Multiple Sclerosis/ |
|  |  |  |  |  |  | Parkinson’s Disease/ |
|  | ‘Motor neuron* disease*’ or MND | | | | | |
|  | ‘Multiple Sclerosis’ or MS | | | | | |
|  | ‘Demyelinating disease*’ | | | | | |
|  | ‘Parkinson* disease’ or PD | | | | | |
|  | Parkinson* | | | | | |

| **Progressive Neurological Disorders** | | | | | | |
| --- | --- | --- | --- | --- | --- | --- |
| **Stroke** | | | | | | |
| **Thesaurus Terms** | **Medline** | **AMED** | **BNI** | **CINAHL** | **EMBASE** | **PsychINFO** |
|  | Stroke/ | Cerebral infarction/ | Stroke/ | Stroke/ | Stroke/ | Cerebrovascular accidents/ |
|  | Brain ischemia/ | Cerebral ischemia/ |  |  |  |  |
|  |  | Stroke/ |  |  |  |  |
|  |  | Cerebrovascular accident/ |  |  |  |  |
|  | (Stroke or poststroke or post-stroke or cerebrovascu* or ‘brain vasc*’ or ‘cerebral vasc*’ or cva* or apoplexy* or sah) | | | | | |
|  | ((brain* or Cerebr* OR vascular OR cerebell* or intracran* or intracerebral* or subarachnoid) ADJ1 (accident OR isch?mi* OR infarct* or thrombo* or emboli* or occlus* or h?morrhage or h?matoma* or bleed*)) | | | | | |

| **Generic LTC** | | | | | | |
| --- | --- | --- | --- | --- | --- | --- |
| **Thesaurus Terms** | **Medline** | **AMED** | **BNI** | **CINAHL** | **EMBASE** | **PsychINFO** |
|  | Chronic disease/ | Chronic disease/ | None found | Chronic disease/ | Chronic disease/ | Chronic pain/ |
|  |  |  |  |  |  | Chronic illness/ |
|  |  |  |  |  |  | Chronicity (disorders)/ |
|  | ((long or chronic or longterm or long-term or long-standing or limiting or ‘non communicable’ or non-communicable or noncommunicable) adj2 (condition* or illness* or disease* or health)) | | | | | |

1.3 Search terms: specific databases

| **BNI** | **CINAHL** |
| --- | --- |
| \| (long or chronic or longterm or long-term or long-standing or limiting or “non communicable” or non-communicable or noncommunicable) ADJ2 condition* \| \| --- \| \| (long or chronic or longterm or long-term or long-standing or limiting or “non communicable” or non-communicable or noncommunicable) ADJ2 illness* \| \| (long or chronic or longterm or long-term or long-standing or limiting or “non communicable” or non-communicable or noncommunicable) ADJ2 disease* \| \| (long or chronic or longterm or long-term or long-standing or limiting or “non communicable” or non-communicable or noncommunicable) ADJ2 health \| | Chronic adj2 condition* |
|  | Chronic adj2 illness* |
|  | Chronic adj2 disease* |
|  | Chronic adj2 health |
|  | Longterm adj2 condition* |
|  | Longterm adj2 illness* |
|  | Longterm adj2 disease* |
|  | Longterm adj2 health |
|  | Long-term adj2 condition* |
|  | Long-term adj2 illness* |
|  | Long-term adj2 disease* |
|  | Long-term adj2 health |
|  | long-standing adj2 condition* |
|  | long-standing adj2 disease* |
|  | long-standing adj2 illness* |
|  | long-standing adj2 health |
|  | Limiting adj2 condition* |
|  | Limiting adj2 disease* |
|  | Limiting adj2 illness* |
|  | Limiting adj2 health |
|  | ‘non communicable’ adj2 condition* |
|  | ‘non communicable’ adj2 disease* |
|  | ‘non communicable’ adj2 illness* |
|  | ‘non communicable’ adj2 health |
|  | Non-communicable adj2 condition* |
|  | Non-communicable adj2 disease* |
|  | Non-communicable adj2 illness* |
|  | Non-communicable adj2 health |
|  | noncommunicable adj2 condition* |
|  | noncommunicable adj2 disease* |
|  | noncommunicable adj2 illness* |
|  | noncommunicable adj2 health |

1.4 Self-management support terms

| **Self-Management Support terms** | | | | |
| --- | --- | --- | --- | --- |
| Thesaurus | **Medline** | **AMED** | **EMBASE** | **PsychINFO** |
|  | Self care/ | Self care/ | Self care/ | Self care skills/ |
|  | (Self ADJ1 (car* or manag* or help or admistrat* or monitor* or medicat*)) or self-car* or self-manag* or self-help or self-adminisrat* or self-monitor* or self-medicat* or selfcar* or selfmanagement or selfhelp or selfadministrat* or selfmonitor* or selfmedicat* | | | |
|  | ‘expert patient’ | | | |
|  | (Tele ADJ2 (health or medicine or care)) or tele-health or tele-medicine or tele-care or telehealth or telemedicine or telecare | | | |
|  | ‘Short message service’ or SMS or ‘mobile phone’ or ‘text message*’ | | | |
|  | ‘Action plan*’ | | | |
|  | ‘personal health budget*’ | | | |
|  | (Peer or patient or emotional or social or psychosocial) ADJ1 (support or group) | | | |

| **BNI** | **CINAHL** |
| --- | --- |
| Self care/ | Self care/ |
| Self ADJ2 (car* or manag* or help or admistrat* or monitor* or medicat*) | Self ADJ2 car* |
|  | Self ADJ2 manag* |
|  | Self ADJ2 help |
|  | Self ADJ2 administrat* |
|  | Self ADJ2 monitor* |
|  | Self ADJ2 medicat* |
| self-car* or self-manag* or self-help or self-adminisrat* or self-monitor* or self-medicat* or selfcar* or selfmanagement or selfhelp or selfadministrat* or selfmonitor* or selfmedicat* | self-car* |
|  | self-manag* |
|  | self-help |
|  | self-adminisrat* |
|  | self-monitor* |
|  | self-medicat* |
|  | selfcar* |
|  | Selfmanagement |
|  | Selfhelp |
|  | selfadministrat* |
|  | selfmonitor* |
|  | selfmedicat* |
| “expert patient” | “expert patient” |
| (Tele ADJ2 (health or medicine or care)) or tele-health or tele-medicine or tele-care or telehealth or telemedicine or telecare | Tele ADJ2 health |
|  | Tele ADJ2 medicine |
|  | Tele ADJ2 care |
|  | tele-health |
|  | tele-medicine |
|  | tele-care |
|  | Telehealth |
|  | Telemedicine |
|  | Telecare |
| “Short message service” or SMS or “mobile phone” or “text message*” | “text message*” |
|  | “Short message service” |
|  | SMS |
|  | “mobile phone” |
| “Action plan*” | “Action plan*” |
| (Peer or patient or emotional or social or psychosocial) ADJ1 support | Peer ADJ1 support |
|  | Patient ADJ1 support |
|  | Emotional ADJ1 support |
|  | Social ADJ1 support |
|  | Psychosocial ADJ1 support |
| (Peer or patient or emotional or social or psychosocial) ADJ1 group | Patient ADJ1 group |
|  | Peer ADJ1 group |
|  | Emotional ADJ1 group |
|  | Social ADJ1 group |
|  | Psychosocial ADJ1 group |
| “Expert patient” | “Expert patient” |
| “personal health budget*” | “personal health budget*” |
