# Supplementary material for: Implementing supported self-management for asthma: a systematic review and suggested hierarchy of evidence of implementation studies
Source: BMC Med. 2015 Jun 1;13:127. doi: 10.1186/s12916-015-0361-0 (PMC4465463; doi:10.1186/s12916-015-0361-0)
Supplement: Additional file 2: — Exclusion criteria. [file 12916_2015_361_MOESM2_ESM.docx]

# Additional file 2. Exclusion criteria

EXCLUDE 1. Exclude if it is not written in English.

EXCLUDE 2. Exclude if not a primary empirical study (do not include if review of literature or conceptual/philosophical papers).

EXCLUDE 3. Exclude if the paper does not focus on, or include one or more of the exemplar LTCs.

EXCLUDE 4. Exclude if the study is not an intervention

EXCLUDE 5: Exclude if the focus is not about SM support interventions.

EXCLUDE 6: Exclude if the paper is NOT an implementation trial (ie delivered as part of routine service to populations of people). Do not include Phase III RCTs or qualitative studies as these are included in the two other PRISMS reviews).

EXCLUDE 7: Exclude if does not measure one of the outcomes of interest (use of healthcare services, health outcomes, symptoms, health behaviour, quality of life or self-efficacy).

EXCLUDE 8: if more than one LTCs included, exclude if unable to data extract the information for the selected LTCs separately from the rest of the findings for the other LCTs.
