# Supplementary material for: Implementing supported self-management for asthma: a systematic review and suggested hierarchy of evidence of implementation studies
Source: BMC Med. 2015 Jun 1;13:127. doi: 10.1186/s12916-015-0361-0 (PMC4465463; doi:10.1186/s12916-015-0361-0)
Supplement: Additional file 3: — Description of studies, participants and service implementation. [file 12916_2015_361_MOESM3_ESM.docx]

**Additional file 3:** **Description of studies, participants and service implementation**

Key to abbreviations in the table: PAAP= Personalised asthma action plan; lTC=Long-term condition; CCM= Chronic Care Model; MCO=Managed Care Organisation, COPD= chronic obstructive pulmonary disease

RCT=randomised controlled trial; I= Intervention group; C=Control group

y, m, w or d = year, month, week or day, hr=hour, min=minute

| **Study,**  **LTC,**  **country and setting** | **Study design** | | **Participants** | | | | | **Service implementation** | |
| --- | --- | --- | --- | --- | --- | --- | --- | --- | --- |
|  | **Design**  **Underpinning evidence, theory** | **Aim** | **Eligibility criteria** | **Total eligible population** | **Number (%) participating** | **Participating population characteristics** | **Attrition** | **Intervention and strategy for implementation** | **Duration, intensity,**  **frequency** |
| **Primarily professional training** | | | | | | | | | |
| **Cleland 2007 [1]**  UK  Primary care | Cluster RCT  Informed by RCTs | To assess the effectiveness in real life primary care of training designed to support practice nurses in implementing PAAPs | 13 practices: using a compatible electronic health record Patients aged 18 to 55y with asthma for >1y receiving regular preventive asthma therapy | Identified as having poor asthma control  667 | Routine data: 629  Questionnaire 177 | No demographic data on total eligible population.  Questionnaire responders were older, more likely to be female, and used fewer beta_2_ agonists and courses of oral steroids than those who consented to use of routine data | No practice attrition.  Poorly controlled asthmatics invited to a) allow access to routine data, and b) complete questionnaires. Non-response at follow up  I = 31 (24%),  C – 28 (26%) | Intervention focused on practice nurse training. Doctors aware, but no attempt to facilitate incorporation into practice procedures.  Professionals’ training: focused on clinical and communication skills, case studies to practise formulation and review of individualised PAAPs, role play with actors with feedback , communication strategies and patient resources. | Duration of follow up: 6m  Intensity and Frequency: 1d, 3hr seminar |
| **Study, LTC**  **and setting** | **Study design** | | **Participants** | | | | | **Service implementation** | |
|  | **Design/theory** | **Aim** | **Eligibility** | **Eligible** | **Participating** | **Characteristics** | **Attrition** | **Intervention and strategy** | **Duration** |
| **Homer 2005 [2]**  US  Primary care | Cluster RCT based on national asthma guidelines, and evidence based behavioural change theory | To implement and test a quality improvement intervention  intended to improve care and outcomes  for patients with childhood asthma | Practices: providing paediatric asthma care. Patients: Children aged 2y to 16y with asthma and using asthma medication in the previous year. | 13,878 | 631 | Mean age: I= 8.5y; C= 9y, Gender: intervention 64% male, control group 60% male | 3 intervention practices withdrew during study year (1 after randomisation)  Attendance at sessions declined progressively. Only 42% of the practices submitted any data.  Patients: 490 of 631 (78%) provided follow-up interviews | A theoretically-based quality improvement intervention, designed to engage and support the practice team to achieve change.  Professional teams (doctor, nurse and administrator) participated in a learning collaborative project to facilitate asthma management based on the CCM. Provision of additional learning tools regular support | Duration: 12 months  3 x 1-day learning sessions + additional learning sessions + biweekly conference calls |
| **Study,**  **LTC,**  **country and setting** | **Study design** | | **Participants** | | | | | **Service implementation** | |
|  | **Design**  **Underpinning evidence, theory** | **Aim** | **Eligibility criteria** | **Total eligible population** | **Number (%) participating** | **Participating population characteristics** | **Attrition** | **Intervention and strategy for implementation** | **Duration, intensity,**  **frequency** |
| **Primarily patient education** | | | | | | | | | |
| **Delaronde** **2005 [3]**  US  Managed Care Organisation | Preference trial (‘opt-in’ ‘opt-out’ ‘probably’ group were randomised, and ‘non-responders’)informed by national guidelines | To assess the effectiveness of an asthma management programme on the Asthma Medication index (ratio of preventer /reliever medication: optimal prescribing is ≥0.50) | Members of the ConnectiCare Inc & Affiliates programme for at least 3m , aged 13 to 65y, excluding those whose asthma medication index was already optimal | 836 | 399  28 ‘opt-in’  52 ‘opt-out’ 67 allocated to intervention, 67 allocated to control  185 ‘non-responders’ | Mean age: 45y, Gender: 51% female | 27% of the intervention group and 68% of the opt-in group completed four or more contacts | Centrally-led initiative. Administration of the scheme and nurse educators funded and undertaken by the MCO.  Intervention: telephone self-management education including review of asthma and asthma knowledge, with feedback about different asthma behaviours and provision of personalised educational materials. | Duration: 6m  Intensity and Frequency: 1 phone call every 6w |
| **Study, LTC**  **and setting** | **Study design** | | **Participants** | | | | | **Service implementation** | |
|  | **Design/theory** | **Aim** | **Eligibility** | **Eligible** | **Participating** | **Characteristics** | **Attrition** | **Intervention and strategy** | **Duration** |
| **Vollmer** **2006 [4]**  US  Managed Care Organisation | RCT based on guidelines | To test the ability of an automated telephone outreach intervention to reduce acute healthcare utilisation and improve quality of life among adult asthma patients in a large MCO | Adults on regular medication, or considered to be at high risk (e.g. because of using ≥ 4 inhalers a year) COPD patients were excluded. | 6,948 (patients were able to opt out of the service) | All patients were randomly allocated to intervention or control  192 given ‘live’ telephone calls from a real person | Mean age 52y (range 18-99y), Gender: 65% female | 38% participated in the first call, 32% in the second, and 18% in the third. Overall, 47% of intervention participants completed at least one intervention call, and 12% completed all 3 calls. | Centrally-led initiative. Recruitment and set-up undertaken by the MCO  Automated telephone calls to patients: assessing asthma control, providing tailored information. Patients at risk of exacerbations, were flagged for the attention of their usual physician. Patients not regularly reviewed were allocated a primary care physician. | Duration: 10m: Frequency & intensity: 3 rounds of calling, lasting 10 mins |
| **Study, LTC**  **and setting** | **Study design** | | **Participants** | | | | | **Service implementation** | |
|  | **Design/theory** | **Aim** | **Eligibility** | **Eligible** | **Participating** | **Characteristics** | **Attrition** | **Intervention and strategy** | **Duration** |
| **Bunting 2006 [5]**  US  Managed Care Organisation | Time series analysis  Based on previous RCTs | To assess the clinical, and economic outcomes of a pharmacist-driven medication therapy management programme for patients with asthma | All patients with a diagnosis of asthma covered by the participating employers’ health plans, and who had accessed at least one aspect of the programme | Not stated | 207 | Mean age: 42y (men 40y; women 42y), Gender: 72% female | 39 had withdrawn by the end of the study period: 30 because of changes in employment and/or insurance status. 9 ‘failed to meet the requirements of the programme’. | Based on a diabetes programme, the insurance-based intervention was financially supported by employers who waived fees for patients who attended the programme.  Patients chose a pharmacist who regularly reviewed medication, inhaler technique, triggers and environmental control and individual asthma education including a PAAP. | Duration: 5y. (Included patients were in the programme for at least a year)  60-90 min sessions, every 3m |
| **Study, LTC**  **and setting** | **Study design** | | **Participants** | | | | | **Service implementation** | |
|  | **Design/theory** | **Aim** | **Eligibility** | **Eligible** | **Participating** | **Characteristics** | **Attrition** | **Intervention and strategy** | **Duration** |
| **Forshee 1998 [6]**  US  Managed Care Organisation | Prospective before & after study with four assessments post-intervention | To evaluate an intensive education programme for high risk patients | People with poorly controlled asthma (age 5-65y) | Not reported | 201 patients from four managed care plans | Mean age = 34y Gender: 74% female | Not reported | The programme consisted of one-to-one nurse-to-patient ("nurse champion") education | Duration: 24w.  Intensity and Frequency: 4 contacts every 6w |
| **Study, LTC**  **and setting** | **Study design** | | **Participants** | | | | | **Service implementation** | |
|  | **Design/theory** | **Aim** | **Eligibility** | **Eligible** | **Participating** | **Characteristics** | **Attrition** | **Intervention and strategy** | **Duration** |
| **Gerald 2006 [7]**  US  Inner city elementary schools | Cluster RCT (the intervention was delivered in a (random) third of schools each year.  Underpinning theory/guidelines not discussed | To evaluate a comprehensive school-based asthma management programme designed to address the high rates of asthma morbidity in African-American children from low income backgrounds. | Children with asthma (previously diagnosed and newly diagnosed by questionnaire + spirometry with exercise test) | 54 elementary schools (total 13,247 pupils) | 736 children with asthma of whom 603 (82%) attended the physician assessment.  610 were still at the same school for the final assessment | 46% female, 97% African-American. Fist to fourth grade pupils (no age given) | Substantial turnover in school register (12% transferred out of the school system and were lost to follow-up: another 5% transferred between immediate and delayed intervention group schools. | Comprehensive programme which provided training for school staff, classroom education for all school children about asthma, and specific group education to students with asthma as well as an individual session with a physician who reviewed medication and developed a PAAP. | Duration: One school year Intensity *Staff:* one in-service training. *School:* 1 x 30min classroom lesson A*sthma children:* 6 x 30 min sessions + one consultation |
| **Study, LTC**  **and setting** | **Study design** | | **Participants** | | | | | **Service implementation** | |
|  | **Design/theory** | **Aim** | **Eligibility** | **Eligible** | **Participating** | **Characteristics** | **Attrition** | **Intervention and strategy** | **Duration** |
| **Chini 2011 [8]**  Italy  Primary schools | Before & after study.  Based on evidence from systematic reviews | To evaluate a comprehensive school-based asthma programme in which healthcare actions beginning at school are followed up with child’s family at home | Schoolchildren with asthma (identified by questionnaire) | 6 primary schools (total population 2,765): 135 with asthma | 135 | 2,765 school children, aged 6–10y. | None reported | A comprehensive asthma programme, based on a strong family–physician–school relationship, provided an educational intervention to families, school staff, and pupils.  Questionnaire identification of children with asthma, individual diagnosis, management, and extracurricular activities | Duration: Set-up in each school 2m, then 7m individual and group work with children with asthma.  2 schools a year participated in the 1-y programme |
| **Study,**  **LTC,**  **country and setting** | **Study design** | | **Participants** | | | | | **Service implementation** | |
|  | **Design**  **Underpinning evidence, theory** | **Aim** | **Eligibility criteria** | **Total eligible population** | **Number (%) participating** | **Participating population characteristics** | **Attrition** | **Intervention and strategy for implementation** | **Duration, intensity,**  **frequency** |
| **Primarily organisational change** | | | | | | | | | |
| **Kemple 2003 [9]**  UK Primary Care | RCT  based on evidence for self-management and regular review | To assess whether a mailed review reminder with a PAAP improves patients’ understanding and use of self-management | Patients ≥16y, due an asthma review, excluding people with COPD | 1209  (663 excluded because not using regular therapy) | 545 | Median age by group:  C: 38y; I^1^=44y; I^2^=43y  Gender: 58%, 60%, 56% in the three groups  Current smokers: 34%, 25%, 26% | N/A | Initiative undertaken at practice organisational level. Apart from the GP author, involvement of other professionals in the practice is not described.  Three groups:  C: mailed invitation for an asthma review  I^1^: invitation enclosing blank PAAP  I^2^: invitation enclosing partially completed PAAP | Single intervention Duration of follow up = 12m |
| **Study, LTC**  **and setting** | **Study design** | | **Participants** | | | | | **Service implementation** | |
|  | **Design/theory** | **Aim** | **Eligibility** | **Eligible** | **Participating** | **Characteristics** | **Attrition** | **Intervention and strategy** | **Duration** |
| **Pinnock 2007 [10]**  UK  Primary Care | Controlled implementation trial  Building on the evidence from a RCT | To evaluate the effectiveness of a telephone option as part of a routine structured asthma review service | All patients on the 'active asthma’ register (defined as patients with a diagnosis of asthma and prescribed asthma medication in the previous year) | 1,809 people with active asthma | 1,809 were eligible for the service. Sub-group of 822 responded to survey | Mean (SD) age. TC 43.0 (24.8), FtF 42.3 (24.4), UC 45.4 (24.1)  Gender. Female, n (%) TC 309 (55.8) FtF 363 (55.1) UC 285 (55.3) | There was a 20% turnover in patients with ‘active asthma’ in the course of the year | Asthma review included assessment of control, adjustment of treatment and provision of self-management.  Three groups:  TC = telephone-option group: 3 reminders option to choose telephone or face-to-face review  FtF - face-to-face only group: 3 reminders face-to-face with no option to choose telephone  UC – no reminders, usual care | Duration: 12m  Intensity: Clinics offered a range of appointment times throughout the week. Telephone calls could be booked or made opportunistically to non-responders |
| **Study, LTC**  **and setting** | **Study design** | | **Participants** | | | | | **Service implementation** | |
|  | **Design/theory** | **Aim** | **Eligibility** | **Eligible** | **Participating** | **Characteristics** | **Attrition** | **Intervention and strategy** | **Duration** |
| **Lindberg 2002 [11]** Sweden  Primary care | Cross sectional audit of routine clinical records + prospective patient survey.  Based on the literature on nurse-led asthma clinics | To compare the care of patients with asthma in a primary health care centre with an asthma nurse practitioner (ANP) with traditional models of delivering asthma care. | Asthma patients ≥7y, who visited the centres during the 3-m period | Prevalence: 4.1% in ANP practice and 2.2% in the 7 control practices | *Records:* random sample of 20/practice.  *Survey response rate:*  ANP centre: 186 (82%) Control centres 161 (53%) | Median age: ANP centre: 55 years; control centres: 51 years  Gender: ANP centre: 53% female, control centre 55% females | N/A  8 patients excluded from the control centres (asthma diagnosis not confirmed)  1 centre excluded from the survey (only one response) | The existing ANP had her own practice with regular patient follow-ups. She provided regular review, patient asthma education including a PAAP, use of inhalers, avoidance of environmental triggers. | The ANP had been in post for 1 year at the time of the study |
| **Study,**  **LTC,**  **country and setting** | **Study design** | | **Participants** | | | | | **Service implementation** | |
|  | **Design**  **Underpinning evidence, theory** | **Aim** | **Eligibility criteria** | **Total eligible population** | **Number (%) participating** | **Participating population characteristics** | **Attrition** | **Intervention and strategy for implementation** | **Duration, intensity,**  **frequency** |
| **A whole systems approach** | | | | | | | | | |
| **Haahtela 2006 [12]**  Finland  Primary, secondary and community settings | Longitudinal evaluation over the 10-y programme.  Interventions were based on international guidelines | To implement a national asthma care programme to improve care of asthma and thus lessen burden to individuals and the society and prevent an increase in costs | All people in Finland with asthma | 225,000 in 1994 rising to 350,000 in 2004 | N/A | All people with asthma in Finland | N/A | The Finnish programme is ‘comprehensive and reaches deep into the structures of health care’ Goals included:   - early diagnosis and active treatment; - guided self-management as the primary form of treatment - Patient education personalised and timely - Increase in asthma knowledge in key group - reduction in respiratory irritants (e.g. smoking) - promotion of research. | Duration: 1994 – 2004; Frequency and duration: On-going programme for all healthcare organisations and professionals.  Actively involved professional bodies, patient organisations |
| **Kauppi 2012 [13]** | This publication reports follow on data from Haahtela 2006 (the Finnish study: see previous entry). All the descriptive information is therefore the same | | | | | | | | |
| **Study, LTC**  **and setting** | **Study design** | | **Participants** | | | | | **Service implementation** | |
|  | **Design/theory** | **Aim** | **Eligibility** | **Eligible** | **Participating** | **Characteristics** | **Attrition** | **Intervention and strategy** | **Duration** |
| **Souza-Machado 2010 [14]**  Brazil  Community | Controlled longitudinal study over 9y  Based on evidence and policy initiatives | To evaluate the impact of a ‘Programme for Control of Asthma’ in the city of Salvador on hospitalisations | Severe asthma as defined by global guidelines | All people in Salvador with severe asthma | 1,895 people registered with the programme (but data are from hospital registries) | Not reported | N/A | A public health intervention project, supported by the Brazilian Ministry of Health, which provided free asthma care including individual and group asthma education, free access to specialist care and medication. | Data over 9y: 4 before and 5 after introduction of the programme |
| **Study, LTC**  **and setting** | **Study design** | | **Participants** | | | | | **Service implementation** | |
|  | **Design/theory** | **Aim** | **Eligibility** | **Eligible** | **Participating** | **Characteristics** | **Attrition** | **Intervention and strategy** | **Duration** |
| **Andrade 2010 [15]**  Brazil Primary healthcare network | Real-life historical cohort study  Informed by national guidelines | To assess the effectiveness of a paediatric asthma management programme (including self-management education) on unscheduled health service utilisation | Children aged 4-15y from low-income families, with history of ≥ 2 exacerbations in the previous year, under a paediatrician | 582 | 470 (80.8%) intervention group and 112 controls | Cases (Salvador): mean age 6.8y. 291 (61%) male  Controls (Recife): mean age 6.6y. 62 (57%) male  Cases were more likely to have persistent asthma | Not reported | City-wide public health initiative: Pharmacists and health workers (training not described) provided self-management education reinforced by a PAAP Regular medication was provided free of charge. Comprehensive educational activities provided for patients and caregivers | Follow up: 12m |
| **Study, LTC**  **and setting** | **Study design** | | **Participants** | | | | | **Service implementation** | |
|  | **Design/theory** | **Aim** | **Eligibility** | **Eligible** | **Participating** | **Characteristics** | **Attrition** | **Intervention and strategy** | **Duration** |
| **Bunik** **2011[16]**  US  Secondary care paediatric clinics | Retrospective before and after study using 4y of routine data  based on quality improvement literature | To determine if the quality improvement intervention was associated with improved asthma related outcomes. | Clinic attendees [2006 -2009] aged >2y with a diagnosis of asthma. Children with co-morbidity were excluded | 1,797 | 1,797 | Age: 90% were under the age of 13y.  Gender: 58% female | No mention of attrition | Quality improvement programme designed by a multidisciplinary team which met bi-weekly. Pre-consultation patient questionnaires, provider alerts, and electronic templates within the health record were combined into a collaborative intervention. | Duration: 6m to implement the project.  Intensity: Monthly professional training + bi-weekly staff meetings |
| **Study, LTC**  **and setting** | **Study design** | | **Participants** | | | | | **Service implementation** | |
|  | **Design/theory** | **Aim** | **Eligibility** | **Eligible** | **Participating** | **Characteristics** | **Attrition** | **Intervention and strategy** | **Duration** |
| **Swanson 2000 [17]**  Scotland  Primary Care | Controlled before and after study  Based on national guidelines | To evaluate the impact of the introduction of a Health Board programme (HBp) of asthma care in general practice | People with asthma between the ages of 2-50y registered with the practice at least since January 1992 | All eligible patients in participating practices | Case note survey of 400 asthma patients + a patient survey of 532 asthma clinic attendees | Mean age: = 20.8y in HBp practices and 21.3 years in non-HBp practices | N/A (routinely collected data). Normal turnover in practices | Professional training in implementing the BTS asthma guidelines (including protocol for asthma assessment, follow-up record cards, peak-flow diaries). | **Duration:** 1991 -1994 |
| **Study, LTC**  **and setting** | **Study design** | | **Participants** | | | | | **Service implementation** | |
|  | **Design/theory** | **Aim** | **Eligibility** | **Eligible** | **Participating** | **Characteristics** | **Attrition** | **Intervention and strategy** | **Duration** |
| **Findley** **2011 [18]**  US  Community day care centres for pre-school children | Before-&-after study.  Based on evidence from similar programmes in other contexts | To evaluate the impact of a comprehensive, multi-layered intervention throughout 2003–2008 | Pre-school children with asthma (identified by questionnaire) | 9,279 children: 5,938 parents completed the baseline assessment of whom 1908 had symptoms consistent with asthma | 1,374 parents participated in at least one educational event.  871 enrolled in the programme | Age: mean 4.1y, Gender: 56% male | Healthcare professionals, centre staff, parents and children were able to participate in any or all of the activities.  Only 249 (29%) of parents completed the final assessment | Multilayered intervention in 31 pre-school day care centres. Offering educational activities to centre staff, parents, and children and Physician Asthma Care Education (PACE) training to the childrens’ physicians | Intensity: *Centre* s*taff* 4-h training. M*entors*: 24-hr ‘train the trainer’ teaching  *Parents*: 2x 1-hr workshops  *Physicians* could attend 4hr PACE |
| **Study, LTC**  **and setting** | **Study design** | | **Participants** | | | | | **Service implementation** | |
|  | **Design/theory** | **Aim** | **Eligibility** | **Eligible** | **Participating** | **Characteristics** | **Attrition** | **Intervention and strategy** | **Duration** |
| **Polivka 2011[19]**  US  Deprived community | Before-&-after study using routine data collected by the service personnel.  Based on a ‘Health Homes’ policy initiative | To evaluate the impact of an urban Healthy Homes intervention that included educational home visits to reduce asthma morbidity in children. | Areas with high levels of asthma, and poor housing Properties with low income families and a child <18y with physician diagnosed asthma. | Not reported | 243 families were recruited, but data on 84. (159 (65.4%) were lost to follow-up) | Mean age of the child = 6.9y (SD 4.8), Gender: 62% males | 159 (65.4%) of the original 243 subjects were lost to follow up. Follow up was better in those with a high school diploma (87% vs 76% p = 0.05) | Environmental assessment, followed by educational home visits for parents of children with asthma designed to support housing remediation work to reduce asthma triggers (such as damp, moulds, cockroaches, air fresheners), provide asthma education and PAAPs. . | Duration: 36m  Intensity: 1 environment assessment,  1 education visit (2-3 hrs)  2 Community outreach worker visits 2 and 6w after education. |

**References**

1. Cleland JA, Hall S, Price D, Lee AJ. An exploratory, pragmatic, cluster randomised trial of practice nurse training in the use of asthma action plans. *Prim Care Respir J* 2007;16:311-318
2. Homer CJ, Forbes P, Horvitz L, Peterson LE, Wypij D, Heinrich P*.* Impact of a quality improvement program on care and outcomes for children with asthma. *Arch Pediatr Adolesc Med* 2005;159:464-469
3. Delaronde S, Peruccio DL, Bauer BJ. Improving asthma treatment in a managed care population. *Am J Manag Care* 2005;11:361-368
4. Vollmer WM, Kirshner M, Peters D, Drane A, Stibolt T, Hickey T, *et al.* Use and impact of an automated telephone outreach system for asthma in a managed care setting. *Am J Manag Care* 2006;12:725-733
5. Bunting BA, Cranor CW. The Asheville Project: long-term clinical, humanistic, and economic outcomes of a community-based medication therapy management program for asthma. *J Am Pharm Assoc* 2006;46:133-147
6. Forshee JD, Whalen EB, Hackel R, Butt LT, Smeltzer PA, Martin J, *et al.* The effectiveness of one-on-one nurse education on the outcomes of high-risk adult and pediatric patients with asthma. *Managed Care Interface* 1998;11:82-92
7. Gerald LB, Redden D, Wittich AR, Hains C, Turner-Henson A, Hemstreet MP, *et al.* Outcomes for a comprehensive school-based asthma management program. *J Sch Health* 2006;76:291-296
8. Chini L, Iannini R, Chianca M, Corrente S, Graziani S, La Rocca M, *et al*. Happy air, a successful school-based asthma educational and interventional program for primary school children. *J Asthma* 2011;48:419-426
9. Kemple T, Rogers C. A mailed personalised self-management plan improves attendance and increases patients' understanding of asthma. *Prim Care Respir J* 2003;12:110-114
10. Pinnock H, Adlem L, Gaskin S, Harris J, Snellgrove C, Sheikh A. Accessibility, clinical effectiveness, and practice costs of providing a telephone option for routine asthma reviews: phase 4 controlled implementation study*. Br J Gen Pract* 2007;57:714-722
11. Lindberg M, Ahlner J, Ekström T, Jonsson D, Möller M. Asthma nurse practice improves outcomes and reduces costs in primary health care. *Scand J Caring Sci* 2002;16:73-78
12. Haahtela T, Tuomisto LE, Pietinalho A, Klaukka T, Erhola M, Kaila M, *et al.* A 10 year asthma programme in Finland: major change for the better. *Thorax* 2006;61:663-670
13. Kauppi P, Linna M, Martikainen J, Mäkelä MJ, Haahtela T. Follow-up of the Finnish Asthma Programme 2000-2010: reduction of hospital burden needs risk group rethinking. *Thorax* 2013;68:292-293
14. Souza-Machado C, Souza-Machado A, Franco R, Ponte EV, Barreto ML, Rodrigues LC, *et al.* Rapid reduction in hospitalisations after an intervention to manage severe asthma. *Eur Respir J* 2010;35:515-521
15. Andrade WC, Camargos P, Lasmar L, Bousquet J. A pediatric asthma management program in a low-income setting resulting in reduced use of health service for acute asthma. *Allergy* 2010;65:1472-1477
16. Bunik M, Federico MJ, Beaty B, Rannie M, Olin JT, Kempe A. Quality improvement for asthma care within a hospital-based teaching clinic. *Academic Pediatrics* 2011;11:58-65
17. Swanson V, Wright S, Power KG, Duncan B, Morgan J, Turner E, *et al*. The impact of a structured programme of asthma care in general practice. *Int J Clin Pract* 2000;54:573-580
18. Findley SE, Thomas G, Madera-Reese R, McLeod N, Kintala S, Andres Martinez R, *et al.* A community-based strategy for improving asthma management and outcomes for preschoolers. *Journal of Urban Health* 2011;88 Suppl 1:S85-S99
19. Polivka BJ, Chaudry RV, Crawford J, Bouton P, Sweet L. Impact of an urban healthy homes intervention. *Journal of* *Environmental Health* 2011;73:16-20
