# Supplementary material for: Implementing supported self-management for asthma: a systematic review and suggested hierarchy of evidence of implementation studies
Source: BMC Med. 2015 Jun 1;13:127. doi: 10.1186/s12916-015-0361-0 (PMC4465463; doi:10.1186/s12916-015-0361-0)
Supplement: Additional file 4: — Summary of the findings of the included studies. [file 12916_2015_361_MOESM4_ESM.docx]

**Additional file 4.** **Summary of the findings of the included studies**

Key to abbreviations in the table: PAAP= Personalised asthma action plan; lTC=Long-term condition; CCM= Chronic Care Model; MCO=Managed Care Organisation,

RCT=randomised controlled trial; I= Intervention group; C=Control group. NS= Not significant. B=Baseline FU=Follow up.

QoL=Quality of Life, MCID=Minimum Clinically Important Difference. ED=Emergency Department

SABA=short acting beta-agonist, ICS= inhaled corticosteroid, LABA=long acting beta-agonist

y, m, w or d = year, month, week or day, hr=hour, min=minute

| **Study,**  **LTC,**  **Country and setting** | | | **Study** | | | | | | **Intervention** | | | | **Outcomes** | | | |
| --- | --- | --- | --- | --- | --- | --- | --- | --- | --- | --- | --- | --- | --- | --- | --- | --- |
|  |  |  | **Design and duration** | | | **Quality score** | | | **Patient** | | **Professional** | **Organisation** | **Health service utilisation** | **Disease control and QoL** | | **Process** |
| **Primarily professional training** | | | | | | | | | | | | | | | | |
| **Cleland 2007 [1]**  UK  Primary care | | | Cluster RCT  FU: 6m  Intervention practices and waiting list controls | | | 24 | | | - | | Intervention: one 3-hr interactive seminar vs. control | - | - | *Routine data:*  SABA use: NS  Oral steroids: NS  *Sub-group*:  QoL: I: 6.49 vs C: 6.33 (p=0.03) [less than MCID of 0.5]  Asthma control: NS | | - |
|  |  |  | Participants: 13 practices.  Adults 18-55yrs with poorly controlled asthma  I: 373, C: 256 | | | | | | **Implementation strategy.** Exclusive focus on professional training: no facilitation of integrating into routine practice  **Fidelity:** Inconsistent coding meant it was not possible to ascertain whether nurses in the intervention arm used PAAPs more than nurses in the control arm. | | | |  |  |  |  |
|  |  |  | **Author’s reflections and lessons learned:**  Inconsistent coding meant routine data were frequently missing in the electronic health record. For example, whether a nurse-led review had occurred, or whether a PAAP had been issued.  A one-off intervention may be insufficient to teach and reinforce behaviour change: the authors comment they had underestimated the complexity of the support required. | | | | | | | | | | | | | |
| **Study, LTC,**  **and setting** | | | **Study** | | | | | | **Intervention** | | | | **Outcomes** | | | |
|  |  |  | **Design** | | | **Quality** | | | **Patient** | | **Professional** | **Organisation** | **Health service use** | **Control and QoL** | | **Process** |
| **Homer 2005 [2]**  US  Primary care | | | Cluster RCT  FU: 12 months | | | 18 | | | - | | Three 1-d group training + 2 additional sessions + biweekly conference calls | Intended implementation of CCM | Admissions: no between group difference  (both groups improved) | Asthma attacks: no between group difference  (both groups improved) | | Ownership of PAAPs I: 54% vs C: 41% (but large baseline difference) |
|  |  |  | 43 practices (13,878 children with asthma).  Sub-group of 631 children provided interviews | | | | | | **Implementation strategy:** A theoretically-based quality improvement intervention, but organisational barriers impeded the process  **Fidelity:** Considerable lack of engagement. E.g. substantial attrition from training, only 42% of practices submitted performance data | | | |  |  |  |  |
|  |  |  | **Author’s reflections and lessons learned:**  Implementation in primary care practices is challenging. Specific organisational barriers encountered included: changing practice networks, financially challenged managed care plans, and changes in staffing and contracts for providers | | | | | | | | | | | | | |
| **Study,**  **LTC,**  **Country and setting** | | | **Study** | | | | | | **Intervention** | | | | **Outcomes** | | | |
|  |  |  | **Design and duration** | | | **Quality score** | | | **Patient** | | **Professional** | **Organisation** | **Health service utilisation** | **Disease control and QoL** | | **Process** |
| **Primarily patient education** | | | | | | | | | | | | | | | | |
| **Delaronde** **2005 [3]**  US  Managed Care Organisation | | | Preference RCT  FU: 12-m  (‘opt-in’ ‘opt-out’ ‘probably’ group were randomised, and ‘non-responders’) | | | 20 | | | 6-m programme of nurse-led individualized telephonic case management  vs usual care | | - | - | There were no significant differences in the numbers of physician office visits, ED visits, or hospitalisations | QoL improved in the intervention group (P=0.04) though not by the MCID, but not in the control group. | | Ratio of preventer/reliever medication improved in all groups. Increase in intervention group (0.176) was twice that in the control group (0.091) p=0.04. The increase in the ‘opt-in’ group was even larger at 0.285 |
|  |  |  | 399 adults not using a recommended asthma medications regime | | | | | | **Implementation strategy:** Administration of the scheme and nurse educators funded and undertaken by the managed care organisation  **Fidelity:** intervention delivered by certified asthma trainers – no description of any quality checks. 27% of the intervention group and 68% of the opt-in group completed four or more contacts | | | |  |  |  |  |
|  |  |  | **Author’s reflections and lessons learned:**  Self-motivation is an important indicator of readiness to initiate and maintain asthma self-management. | | | | | | | | | | | | | |
| **Study, LTC,**  **and setting** | | | **Study** | | | | | | **Intervention** | | | | **Outcomes** | | | |
|  |  |  | **Design** | | | **Quality** | | | **Patient** | | **Professional** | **Organisation** | **Health service use** | **Control and QoL** | | **Process** |
| **Vollmer** **2006 [4]**  US  Managed Care Organisation | | | RCT  3,581 telephone outreach.  3,367 Usual care | | | 18 | | | Three 10-min automated calls providing asthma review and personalised feedback  (192 had live calls) | | - | Provided as a service by the MCO | Healthcare utilisation: No difference in proportion of patients with unscheduled care (I=10.9 vs C=10.00. p=0.14), or proportion attending ED/admission (I=4.1 vs C=4.0. p=0.88), | Asthma control: No difference in QoL (p=0.56) or asthma symptoms | | Medication use: No difference in ICS use (p=0.20) or SABA use (p=0.86)  [In a post-hoc per-protocol analysis, intervention patients were more likely to received ICS and less to request SABA] |
|  |  |  | 6,948 people with asthma. | | | | | | Implementation strategy: Recruitment and set-up undertaken by the MCO  Fidelity: automated telephone call. 47.0% of intervention participants completed at least 1 intervention call, and 12.1% completed all 3 calls  59.9% of live-caller participants completed at least 1 call and 27.6% completed all 3 calls vs. automated-caller arm were 46.3% and 11.2% respectively. | | | |  |  |  |  |
|  |  |  | **Author’s reflections and lessons learned:**  Common reasons for nonparticipation were unwillingness to talk to a computer, lack of time, and “my asthma is already well controlled”  A more focused intervention (such as promoting medication adherence in patients who have not refilled a prescription for preventer medication) might be more effective. | | | | | | | | | | | | | |
| **Study, LTC,**  **and setting** | | | **Study** | | | | | | **Intervention** | | | | **Outcomes** | | | |
|  |  |  | **Design** | | | **Quality** | | | **Patient** | | **Professional** | **Organisation** | **Health service use** | **Control and QoL** | | **Process** |
| **Bunting 2006 [5]**  US  Managed Care Organisation | | | Time series analysis.  Routinely collected outcomes at annual intervals over 1 to 5y | | | 17 | | | One-to-one education (60-90min sessions) + PAAP by a hospital based asthma educator + regular follow-up for 5y by pharmacists. | | - | Pharmacist costs reimbursed by health plans. Waived medication co-payments on asthma-related medications | *From insurance claims:* Hospitalisations/ED attendance rates for the 3 years before enrolment were much higher at 21.3, 22.2, 22.3 events/100 patients/y than during the 5y of the programme, which averaged 5.4, 2.6, 1.9, 5.4, 0 events/100patients/y. | Compared to baseline, at most recent follow up the proportion of patents with:  • severe /moderate asthma reduced (82% to 49%).  • normal FEV_1_ increased (50% to 75%)  • night time waking: reduced (28% to 12%)  • exacerbations: reduced (35% to 16%)   - Working days lost reduced (2.5 to 0.5 d/y) | | PAAP ownership increased from 63% at baseline to 99% at follow-up (p<.0001) |
|  |  |  | 207 adults covered by employer’s  health plans | | | | | | **Implementation strategy:** The programme was implemented through an employers’ insurance-based scheme. The cost to the employers of providing financial incentives was offset by the reduction in absenteeism.  **Fidelity:** unclear. 9 patients were reported as dropping out for ‘failure to meet the requirements of the programme’ | | | |  |  |  |  |
|  |  |  | **Author’s reflections and lessons learned:**  There was no attempt to determine the contribution of the various interventions (asthma education, long-term follow-up, financial incentives): the authors concluded that a combination of these factors led to the observed outcome improvements | | | | | | | | | | | | | |
| **Study, LTC,**  **and setting** | | | **Study** | | | | | | **Intervention** | | | | **Outcomes** | | | |
|  |  |  | **Design** | | | **Quality** | | | **Patient** | | **Professional** | **Organisation** | **Health service use** | **Control and QoL** | | **Process** |
| **Forshee 1998 [6]**  US  Managed Care Organisation | | Before-&-after study. FU: 24w  4 assessments | | | 15 | | | Tailored individualised education + videos + handouts | | | Nurse champions were educated about asthma | - | Compared to baseline, at follow up over 24 weeks adults and children had:   - Fewer exacerbations: p≤0.01 - Fewer urgent visits (p≤0.01) | Compared to baseline, at follow up over 24w adults and children had:   - Improved QoL (p≤0.01) - Fewer days off work [B: 6.5d vs FU: 3.9d (p≤0.001)] | | Monthly reviews increased for both adult and children (p≤0.05) and specialist referrals (p≤0.05) |
|  |  | 201 adults and children with poorly controlled asthma from four MCOs | | | | | | **Implementation strategy:** training of a nurse champion  **Fidelity:** No information | | | | |  |  |  |  |
|  |  | **Author’s reflections and lessons learned:**  Training an existing employee to deliver the intervention enabled intervention implementation compared to training a contractor who did not already have a relationship with the patients. | | | | | | | | | | | | | | |
| **Study, LTC,**  **and setting** | | | **Study** | | | | | | **Intervention** | | | | **Outcomes** | | | |
|  |  |  | **Design** | | | **Quality** | | | **Patient** | | **Professional** | **Organisation** | **Health service use** | **Control and QoL** | | **Process** |
| **Gerald 2006 [7]**  US  Inner city elementary schools | | Cluster RCT with immediate and delayed groups | | | 18 | | | 6 x 30 min group education for pupils with asthma + a consultation with a paediatric allergist confirmed the diagnosis, reviewed medication and provided a PAAP | | | .- | Asthma education was provided for school staff in an ‘in-service’ meeting. A 30 minute classroom lesson was given to all children in grades I to IV in the school | Compared to baseline, at the end of the school year children had:   - No difference in number of ED visits or hospitalisations   [Note: problems with accuracy of tracking hospital attendances due to multiple registration details and incorrect social security numbers ] | Compared to baseline, at the end of the school year children had:   - No difference in absenteeism   [Note: problems with accuracy of school attendance registers due to different methods of recording partial attendance and concurrent attendance incentive schemes] | | Compared to baseline, post-school education there was a statistically significant increase in knowledge (p <0.0001) |
|  |  | 736 children with asthma from 54 schools. Due to attrition (e.g. moving schools) data on 610 were available for the final analysis | | | | | | **Implementation strategy:** A comprehensive collaboration between school and health services  **Fidelity:** There was significant difficulty in maintaining the fidelity of the programme: students frequently changed schools, high turnover of school staff, workload prevented provision of in-school education, trained volunteer staff proved costly to supervise, education sessions were shortened to save time. | | | | |  |  |  |  |
|  |  | **Author’s reflections and lessons learned:**  Connecting children with a regular source of health care in this inner city African-American population was difficult. More intensive methods of medical management, such as school-based health centres or supervised asthma therapy, might prove more effective in inner-city schools. | | | | | | | | | | | | | | |
| **Study, LTC,**  **and setting** | | | **Study** | | | | | | **Intervention** | | | | **Outcomes** | | | |
|  |  |  | **Design** | | | **Quality** | | | **Patient** | | **Professional** | **Organisation** | **Health service use** | **Control and QoL** | | **Process** |
| **Chini 2011 [8]**  Italy  Primary schools | | | Before-&-after study.  2 schools participated each year in the 1yr programme | | | 15 | | | Children with asthma had a clinical assessment and were given a PAAP with follow-up review at 1y..  Age-appropriate groups taught cognitive techniques and breathing control | | - | Lessons aimed at teachers, school personnel, parents, and schoolchildren to improve their knowledge of asthma | Not assessed | Compared to baseline, at the end of school year:   - Asthma symptom score improved [Mean (SD) B: 86.1 (6.5) vs FU 94.9 (6.1) p<0.001] - QoL improved [mean (SD) B:2.2 (0.79) vs FU 3.5 (0.73) p<0.001] - Parents’ perception of child’s QoL improved [Mean(SD) B: 3.1 (0.6) vs 3.5 (0.4) p=0.004] | | Not assessed |
|  |  |  | 2,765 school children, aged 6–10y. 135 with asthma | | | | | | **Implementation strategy:** A comprehensive asthma programme based on an existing strong family–physician–school relationship  **Fidelity:** No data on attendance at groups, uptake of assessments. | | | |  |  |  |  |
|  |  |  | **Author’s reflections and lessons learned:**  The programme also encouraged a close relationship between families, children, school staff, and physicians through numerous meetings and lessons addressed to all participants and represent a model for combining health care with social care forming a “health-care chain” system | | | | | | | | | | | | | |
| **Study,**  **LTC,**  **Country and setting** | | **Study** | | | | | | **Intervention** | | | | | **Outcomes** | | | |
|  |  | **Design and duration** | | | **Quality score** | | | **Patient** | | | **Professional** | **Organisation** | **Health service utilisation** | **Disease control and QoL** | | **Process** |
| **Primarily organisational change** | | | | | | | | | | | | | | | | |
| **Kemple 2003 [9]**  UK Primary Care | | RCT - 3 groups:  C: Mailed invitation  I^1^: + blank PAAP,  I^2^: + PAAP with patient’s details completed | | | 20 | | | - | | | - | Organisational intervention enclosing blank/partially completed PAAPs with invitations to review | There were no significant differences in use of healthcare resources over the subsequent 12m | There were no significant differences in prescriptions of SABA over the subsequent 12m | | Proportion attending for an asthma review increased (C: 70%, I^1^: 82%, I^2^: 84%. p=0.002)  OR 2.33, 95% CI 1.37-3.93  Personalised plan (I^2^) increased knowledge of using a PAAP (OR 2.58 (95%CI 1.24 to 5.36) |
|  |  | 545 with asthma on regular medication | | | | | | **Implementation strategy:** Administration was at organisational level. Involvement of other professionals in the practice is not described.  **Fidelity:** Content of mailing were controlled but no check on content of the subsequent review | | | | |  |  |  |  |
|  |  | **Author’s reflections and lessons learned:**  Personalised prompts can help, but many patients only respond to prompts and access help when they realise they have a problem | | | | | | | | | | | | | | |
| **Study, LTC,**  **and setting** | | **Study** | | | | | | **Intervention** | | | | | **Outcomes** | | | |
|  |  | **Design** | | | **Quality** | | | **Patient** | | | **Professional** | **Organisation** | **Health service use** | **Control and QoL** | | **Process** |
| **Pinnock 2007 [10]**  UK  Primary Care | Controlled implementation trial over 1y comparing telephone option (TC) with face-to-face only (FtF), and a ‘usual care’ group (UC) | | | | 21 | | | Asthma review according to clinical need, including assessment of control, adjustment of treatment and provision (or review) of self-management (with PAAP). | | | Existing asthma nurses who had an accredited diploma on asthma care and were already providing asthma care in the practuce | Three reminders to patients due a review.  Option in intervention group to book a telephone or face-to-face review. Opportunistic telephone calls to non-responders. | - | Compared to the FtF only group, patients in the TC option group had   - no difference in asthma control - no difference in asthma QoL | Routine asthma review was provided for 397/598 (66.4%) in the TC group and 352/654 (53.8%) in the FtF group [risk difference 12.6% (95% CI 7.2 to 17.9) p<0.001]  Compared to the FtF group, patients in the TC group had:   - increased enablement: [TC: 7.29 (SD 4.26) vs FtF: 6.43 (SD 4.30) p=0.03] - greater confidence in managing asthma (p = 0.007). | |
|  | Routine data from 1,809 people with ‘active asthma’.  Sub-group of 822 responded to survey | | | | | | | **Implementation strategy:** Implemented within the context of the newly introduced Quality and Outcome framework which rewarded the provision of routine asthma reviews. This provided an incentive for the practice to send out reminders and undertake opportunistic telephone calls. Senior practice staff were involved in the design of the interventions.  **Fidelity:** Standardised training was provided for all administrative and nursing staff. The process was monitored on a monthly basis to assess the number of reviews undertaken, and the content of the review (including provision of self-management education | | | | |  |  |  |  |
|  | **Author’s reflections and lessons learned:**  The study coincided with the introduction of the UK GP contract (2004) which rewards practices who achieve clinical standards, including a target of 70% for the annual review of people with asthma.  The data suggest that opportunistic calls should be provided for enhancing access rather than accepting non-response as an exclusion criterion. | | | | | | | | | | | | | | | |
| **Study, LTC,**  **and setting** | | **Study** | | | | | | **Intervention** | | | | | **Outcomes** | | | |
|  |  | **Design** | | | **Quality** | | | **Patient** | | | **Professional** | **Organisation** | **Health service use** | **Control and QoL** | | **Process** |
| **Lindberg 2002 [11]** Sweden  Primary care | Cross sectional audit of routine clinical records + prospective patient survey | | | | 16 | | | The ANP provided regular review, patient asthma education including a PAAP, use of inhalers, avoidance of environmental triggers | | | - | With the exception of emergency visits and the yearly follow-up visit to their physician all reviews were done by the ANP | *Survey* Patients from ANP centre had:   - Lower proportion of emergency GP consultations (4.9% of all visits vs 12.8% in the control centres (p<0.05)) - 18% lower total healthcare costs (38% lower inpatient costs and 35% outpatient costs)   . | *Survey* Patients from ANP centre were less likely to:   - wake at night (p<0.01) - have activity limitation (p<0.05) - have ≥2 asthma attacks in 6m (p<0.05)   ANP centre patients had:   - No difference in health status (Eq5D) - 7% higher indirect costs due to sick leave. | *Clinical records:* ANP centre was more likely to:   - record a peak flow - teach home PF monitoring (p<0.001) - discuss smoking   *Survey:* ANP patients were more likely to:   - own a PAAP (p<0.001) - use a PF meter - have adequate knowledge about asthma (p<0.001) - attend for an asthma check-up (p< 0.001) | |
|  | 1 Asthma Nurse Practitioner (ANP) service + 7 similar practices with no ANP service.  *Records:* random sample of 20 per practice.  *Survey:* ANP centre: 186 (82%) responses. Control centres 161 (53%) responses | | | | | | | **Implementation strategy:** This was an evaluation of an existing service. The ANP had been providing the majority of routine asthma care in one of the primary health care centres for at least 1y  **Fidelity:** N/A as this was an existing service | | | | |  |  |  |  |
|  | **Author’s reflections and lessons learned:**  ANP strategy in primary care can improve asthma care and has economic advantages. However the result may only be generalised to other practices working with asthma nurses in the same way. | | | | | | | | | | | | | | | |
| **Study,**  **LTC,**  **Country and setting** | | **Study** | | | | | | **Intervention** | | | | | **Outcomes** | | | |
|  |  | **Design and duration** | | | **Quality score** | | | **Patient** | | | **Professional** | **Organisation** | **Health service utilisation** | **Disease control and QoL** | | **Process** |
| **A whole systems approach** | | | | | | | | | | | | | | | | |
| **Haahtela 2006 [12]**  Finland  Primary, secondary and community settings | Longitudinal evaluation using national data over the 10-y programme | | | | 10  [Note: many of the criteria did not apply] | | | Patient organisations arranged direct patient counselling and distributing information and resources free of charge | | Education was provided for 5,300 respiratory specialists, 3,700 primary/secondary care professionals, 25,500 other healthcare professionals, 695 pharmacists | | The Ministry of Social Affairs and Health recognised asthma as an important public health issue and set up the national programme  Finnish healthcare was committed to the project | Over the 10 year programme:   - Admissions fell (from 110,000 to 51,000/y) - Deaths fell from 123 to 85/y - ED visits fell - Costs fell (from €1611 per patient to €1031 per patient) | Over the 10 year programme:   - Compensation for lost work days decreased (from 2966 sickness periods to 1920) - The number of people with asthma receiving disability payments decreased (from 7212 to 1741) | Over the 10 year programme:   - Number of asthmatics increased (from 225,000 to 350,000) - Proportion of patients using ICS increased from 33% to 85% - Smoking levels remained constant, but smoking bans have reduced passive exposure | |
|  | All people with asthma in Finland | | | | | | | **Implementation strategy:**. The Finnish programme is ‘comprehensive and reaches deep into the structures of health care’  **Fidelity:** A network of asthma coordinators (a doctor and nurse in each healthcare centre) promoted the initiative and monitored quality | | | | |  |  |  |  |
|  | **Author’s reflections and lessons learned:**  1. a broad commitment by the healthcare system and society is mandatory and should be sought at an early stage. Political commitment is also essential,  2. good asthma management requires multidisciplinary team working and collaboration with the society for early detection and asthma management,  3. professional follow-up and regular evaluation are important to keep the programme running and adjusting to required changes,  4. All professionals involved in asthma care should be trained around asthma self-management to help patients manage their asthma. | | | | | | | | | | | | | | | |
| **Kauppi 2012 [13]** | This publication reports follow on data from the Haahtela Finnish study (see previous entry). All the descriptive information is therefore the same | | | | | | | | | | | | In the 6 years after the end of the programme admissions continued to fall (from 5,894 patients using 32,000 bed days to 2,938 patients using 15,000 bed days) |  | In the 3 years after the end of the programme)   - Prevalence of asthma continued to rise (from 6.8% to 9.4%) | |
| **Study, LTC,**  **and setting** | | **Study** | | | | | | **Intervention** | | | | | **Outcomes** | | | |
|  |  | **Design** | | | **Quality** | | | **Patient** | | | **Professional** | **Organisation** | **Health service use** | **Control and QoL** | | **Process** |
| **Souza-Machado 2010 [14]**  Brazil  Community | Controlled longitudinal study over 9y (4y before and 5y after start of the programme) | | | 11  [Note: many of the criteria did not apply] | | | | | Patient training: individual asthma education + monthly group sessions discussing asthma prevention and treatment | Experienced staff trained 512 primary healthcare physicians, nurses, pharmacists, social workers and managers on the prevention and management of asthma and rhinitis | | Healthcare community project. Centres offered specialist care and free medication to patients with severe asthma | Over the 9y of data collection:   - Asthma admissions fell by 82.3% [from 12.72 to 2.25 per 10,000 inhabitants (p=0.001)] - Hospitalisation in children fell by 78.6% (p=0.001) - Hospitalisation in people >10yrs fell by 93.7% (P=0.001).   The decline was greater in Salvador than in, Recife. (p<0.001) | In-hospital mortality which had been rising from 1998 to 2002, decreased after introduction of the programme from 23 deaths in 2003 to only one in 2006.  (In Recife the in-hospital mortality rate increased from 5 deaths in 2003 to 6 in 2006) | | From 2003 to 2006, the programme dispensed 220,889 units of inhaled medication for asthma control (ICS and/or SABA/LABA). There was a strong inverse correlation between hospitalisation rates and drug dispensation (-0.801;  p=0.001) |
|  | 1,895 people enrolled with the programme (but data are at population level using routine hospital registries)  Data from Salvador were compared with Recife a similar Brazilian city | | | | | | | **Implementation strategy:** The major role of the programme was to catalyse an initiative that combines contributions of all levels of public health administration (city, state and Ministry of Health) into a programme that involves healthcare, capacity building and research.  **Fidelity:** not reported | | | | |  |  |  |  |
|  | **Author’s reflections and lessons learned:**  We speculate that the most important factors that determined our favourable results were: 1) a considerable proportion of patients with severe asthma with no access to good quality care, including affordable medication at baseline; 2) establishment of treatment with the most effective and safe medications; 3) an education programme to increase compliance to medication use and 4) a referral system made easy for patients and the health services | | | | | | | | | | | | | | | |
| **Study, LTC,**  **and setting** | | **Study** | | | | | | **Intervention** | | | | | **Outcomes** | | | |
|  |  | **Design** | | | **Quality** | | | **Patient** | | | **Professional** | **Organisation** | **Health service use** | **Control and QoL** | | **Process** |
| **Andrade 2010 [15]**  Brazil Primary healthcare network | | Historical cohort  FU: 12m | | | 19 | | | Individual and group educational activities, including PAAP | | | Patient education provided by pharmacists and health workers but no details of their training. | Healthcare community project. Free medication | Health service use. At 12m 23/470 (5%) of cases used the Municipal Health System for acute asthma compared to 38/112 (34%) of controls p<0.01 | **-** | | The use of ICS was greater in cases than controls: 68% of cases were ICS users.  All users of the service had a PAAP |
|  |  | 582 children aged 4-15yrs (470 cases, 112 controls)  [Cases were slightly younger and more severe than controls] | | | | | | **Implementation strategy:** City-wide public health initiative: no description of the process or who was involved.  **Fidelity:** All cases had accessed the programme at least once and had been given a PAAP. No information on subsequent attendances. For cost reasons unlikely to have sought medical attention / prescriptions elsewhere | | | | |  |  |  |  |
|  |  | **Author’s reflections and lessons learned:**  Asthma programmes in low-middle income settings should provide anti-asthmatic reliever and controller medicines (free, when possible) as well as a comprehensive educational framework because of their synergistic effect in reducing the burden on health services by acute asthma | | | | | | | | | | | | | | |
| **Study, LTC,**  **and setting** | | **Study** | | | | | | **Intervention** | | | | | **Outcomes** | | | |
|  |  | **Design** | | | **Quality** | | | **Patient** | | | **Professional** | **Organisation** | **Health service use** | **Control and QoL** | | **Process** |
| **Bunik** **2011 [16]**  US  Secondary care paediatric clinics | | Retrospective study using routine data from before, the year before and 4 years after the intervention | | | 15 | | | Asthma educators provided education about medications and devices, and assisted in creating of PAAPs  Telephone calls 2 weeks after any episodes of unscheduled care | | | Monthly 1hr education sessions for junior medical staff and nurses. Familiarisation with the project for all new staff.  Computer and paper prompts to facilitate structured review, appropriate prescribing and provision of PAAPs | Organisational strategies included pre-consultation questionnaires for families, paper alerts and computer templates for asthma reviews, respiratory therapist support for providing education and PAAPs. | There was a trend toward fewer emergency department visits and hospitalisations from 2006 to 2009, but this was not statistically significant. | **_-_** | | Compared to those seen in 2006, children seen 2y after the intervention were more likely to:   - be given a PAAP [RR 2.86, 95% CI 2.60–3.20] - have a recorded assessment of severity [RR 1.47, 95% CI 1.41–1.54) - be prescribed preventer therapy [RR 1.11, 95% CI 1.05–1.19] |
|  |  | 1797 clinic attendees over 4y | | | | | | **Implementation strategy:** Quality improvement programme designed by a multidisciplinary team which met bi-weekly throughout the 6m projecte.  **Fidelity:** Process outcomes (such as provision of PAAPs suggests that protocols were followed, though the volume of patients seen meant that some strategies such as completion of the pre-consultation forms did not always occur | | | | |  |  |  |  |
|  |  | **Author’s reflections and lessons learned:**  Collaborative practice change can be achieved with demonstrable and sustainable effects on important outcomes, but need allocated time for regular meetings and working through several plan/do/study/act cycles while addressing implementation problems | | | | | | | | | | | | | | |
| **Study, LTC,**  **and setting** | | **Study** | | | | | | **Intervention** | | | | | **Outcomes** | | | |
|  |  | **Design** | | | **Quality** | | | **Patient** | | | **Professional** | **Organisation** | **Health service use** | **Control and QoL** | | **Process** |
| **Swanson 2000 [17]**  Scotland  Primary Care | Controlled before and after study | | | 16 | | | Asthma self-management education in asthma clinic | | | Professional training in implementing the national asthma guideline | | Provision of paper-based templates | Compared to control practices, at follow-up patients in HBp practices were:   - Less likely to have had an admission (p<0.05) | Compared to patients in control practices, patients reviewed in HBp practices reported:   - greater improvements in markers of control (time off work or school, sleep disturbances, early morning symptoms) (p<0.001) | | Compared to control practices, at FU patients in HBp practices were:   - More likely to have PAAPs (p<0.01) - Follow their PAAP (p<0.05) - More likely to have attended an asthma review (p<0.005)   PAAP use increased significantly from baseline to FU in HBp practices |
|  | 400 people with asthma (2- 50 years) registered with the practice since 1992 + 532 responders to the survey of asthma clinical attendees | | | | | | **Implementation strategy:** Health Board programme (HBp)  **Fidelity:** Findings report adherence to guidelines as judged by retrospective audit of clinical records | | | | | |  |  |  |  |
|  | **Author’s reflections and lessons learned:**  Some practices were reluctant to take part in the study which may have reflected poor standards of asthma care, whilst those who participated might represent an overoptimistic picture of asthma care. Similarly, patients responding to the survey were those attending the asthma clinics likely to hold more positive perceptions of clinics and proactive care than non-attendees. | | | | | | | | | | | | | | | |
| **Study, LTC,**  **and setting** | | **Study** | | | | | | **Intervention** | | | | | **Outcomes** | | | |
|  |  | **Design** | | | **Quality** | | | **Patient** | | | **Professional** | **Organisation** | **Health service use** | **Control and QoL** | | **Process** |
| **Findley** **2011 [18]**  US  Community day care centres for pre-school children | | Before-&-after study in 35 pre-school centres | | | 17 | | | Parent education workshops: delivered by mentors (parents who manage their children’s asthma well). Parents received education and a PAAP, and were encouraged to talk with their child’s physicians  Children played activities and games on asthma triggers and signs | | | Professionals of children enrolled in the programme were offered Physician Asthma Care Education (PACE) training | The centre staff received training on asthma and asthma management (including creating an ‘asthma-friendly centre’), identifying children with asthma, following a PAAP and handling emergencies | Compared to baseline, at the 9-12m assessment the proportion of children with:   - Hospitalisations reduced from 24% to 11% (p<0.001) - No ED visits increased from 25% to 53% (p<.001).   The greatest benefits were in children whose parents, and physicians engaged in the intervention compared to those receiving ‘Centre-only’ components | Compared to baseline, at the 9-12m assessment the proportion of children with:   - Day-care absences reduced from 56% to 38% - No night-symptoms increased from 19% to 52% (p<0.001) - No day symptoms: increased from 22% to 59% (p<0.001)   The impact was significantly greater in children whose physician attended training (p=0.036) | | Compared to baseline, at the 9-12m assessment:   - PAAP use increased from 47% to 70% - Staff knowledge increased from 49% to 82% - Parents’ knowledge increased from 62% to 79%; - Parents’ confidence in managing their child’s asthma increased from 57% to 81% (p<0.001); |
|  |  | 1,374/1,908 (72%) parents participated in at least one educational event. 871 enrolled in the programme. 249 completed the final assessment | | | | | | **Implementation strategy:** A multi-layered approach as day care centre staff made the centres more asthma-friendly, the programme and centre staff educated parents about asthma management, and the community's paediatric care providers received training to provide state-of-the-art care  **Fidelity:** 31/35 centres completed training and engaged in at least one activity. 871 (46%) of parents enrolled in the programme. 183 (59%) of the childrens’ healthcare providers attended PACE training, No reported assessment on how interventions were implemented | | | | |  |  |  |  |
|  |  | **Author’s reflections and lessons learned:**  A multi-layered approach can improve asthma outcomes among pre-schoolers with a combination of parent and provider education having the greatest impact.  A key element of the strategy was linking asthma education activities in the daycare setting with improving the quality of asthma care by healthcare providers. | | | | | | | | | | | | | | |
| **Study, LTC,**  **and setting** | | **Study** | | | | | | **Intervention** | | | | | **Outcomes** | | | |
|  |  | **Design** | | | **Quality** | | | **Patient** | | | **Professional** | **Organisation** | **Health service use** | **Control and QoL** | | **Process** |
| **Polivka 2011 [19]**  US  Deprived community | Before-&-after study over 2y using routine anonymised data collected by the service personnel | | | 18 | | | Environmental assessment, home repairs, educational home visits to reduce asthma triggers (such as damp, moulds, cockroaches, air fresheners), and provide asthma education and PAAPs | | | Professionals completed the National Centre for Healthy Homes practitioners’ course and the American Lung Association of Ohio’s asthma educator course. | | Costs included repair work, contractors, supplies for assessment and education provided to participants | Compared to baseline, at 2y FU children had:   - fewer emergency health care visits in 3m [B: 1.7 (SD 2.7) vs FU: 0.4 (SD 0.7) (p<0.001)] - Similar hospitalisations in 3m [B: 0.3 (SD1.3) vs FU: 0.09 (SD 0.5) p=0.229 | Compared to baseline, at 2y FU children had:   - fewer days with symptoms/2w [B:5.0 (SD 4.2) vs FU: 2.2 (SD 2.9) (p<0.001)] - fewer nights with symptoms/2w [B: 3.6 (SD 4.1) vs FU 1.8 (SD 3.1) (p<0.001)] - fewer days with activity limitation/2w [B: 4.1 (SD4.6) vs FU:1.7 (SD 3.0) (p<0.001)] - Fewer school days missed/6m [B: 5.9 (SD16.8) vs FU:1.1 (SD 2.4) P <0.001] | | Compared to baseline, at 2y FU:   - asthma knowledge had increased (p<0.001) - The proportion of children with a - PAAP ownership increased [B: 44% → 67% (p=0.007)] - Proportion of children exposed to passive smoking reduced [90% → 70% (p=0.003)] |
|  | 243 families were recruited, 84 (35%) completed | | | | | | **Implementation strategy:** A comprehensive home visitation intervention programme delivered by a multidisciplinary team (programme manager, health educator, sanitarians, community outreach workers) and which provided practical remediation for unhealthy housing as well as asthma health education.  **Fidelity:** Barriers: many clients were highly transient: their housing arrangements or status changed unexpectedly from month to month. | | | | | |  |  |  |  |
|  | **Author’s reflections and lessons learned:**  The cost of interventions averaged $1,242 per unit not including start-up, staffing, training, and overhead costs. These cost considerations point to the difficulty in sustaining efforts like these without grant funding or other support. A related sustainability issue is the on-going need for home maintenance and repair | | | | | | | | | | | | | | | |

**References**

1. Cleland JA, Hall S, Price D, Lee AJ. An exploratory, pragmatic, cluster randomised trial of practice nurse training in the use of asthma action plans. *Prim Care Respir J* 2007;16:311-318
2. Homer CJ, Forbes P, Horvitz L, Peterson LE, Wypij D, Heinrich P*.* Impact of a quality improvement program on care and outcomes for children with asthma. *Arch Pediatr Adolesc Med* 2005;159:464-469
3. Delaronde S, Peruccio DL, Bauer BJ. Improving asthma treatment in a managed care population. *Am J Manag Care* 2005;11:361-368
4. Vollmer WM, Kirshner M, Peters D, Drane A, Stibolt T, Hickey T, *et al.* Use and impact of an automated telephone outreach system for asthma in a managed care setting. *Am J Manag Care* 2006;12:725-733
5. Bunting BA, Cranor CW. The Asheville Project: long-term clinical, humanistic, and economic outcomes of a community-based medication therapy management program for asthma. *J Am Pharm Assoc* 2006;46:133-147
6. Forshee JD, Whalen EB, Hackel R, Butt LT, Smeltzer PA, Martin J, *et al.* The effectiveness of one-on-one nurse education on the outcomes of high-risk adult and pediatric patients with asthma. *Managed Care Interface* 1998;11:82-92
7. Gerald LB, Redden D, Wittich AR, Hains C, Turner-Henson A, Hemstreet MP, *et al.* Outcomes for a comprehensive school-based asthma management program. *J Sch Health* 2006;76:291-296
8. Chini L, Iannini R, Chianca M, Corrente S, Graziani S, La Rocca M, *et al*. Happy air, a successful school-based asthma educational and interventional program for primary school children. *J Asthma* 2011;48:419-426
9. Kemple T, Rogers C. A mailed personalised self-management plan improves attendance and increases patients' understanding of asthma. *Prim Care Respir J* 2003;12:110-114
10. Pinnock H, Adlem L, Gaskin S, Harris J, Snellgrove C, Sheikh A. Accessibility, clinical effectiveness, and practice costs of providing a telephone option for routine asthma reviews: phase 4 controlled implementation study*. Br J Gen Pract* 2007;57:714-722
11. Lindberg M, Ahlner J, Ekström T, Jonsson D, Möller M. Asthma nurse practice improves outcomes and reduces costs in primary health care. *Scand J Caring Sci* 2002;16:73-78
12. Haahtela T, Tuomisto LE, Pietinalho A, Klaukka T, Erhola M, Kaila M, *et al.* A 10 year asthma programme in Finland: major change for the better. *Thorax* 2006;61:663-670
13. Kauppi P, Linna M, Martikainen J, Mäkelä MJ, Haahtela T. Follow-up of the Finnish Asthma Programme 2000-2010: reduction of hospital burden needs risk group rethinking. *Thorax* 2013;68:292-293
14. Souza-Machado C, Souza-Machado A, Franco R, Ponte EV, Barreto ML, Rodrigues LC, *et al.* Rapid reduction in hospitalisations after an intervention to manage severe asthma. *Eur Respir J* 2010;35:515-521
15. Andrade WC, Camargos P, Lasmar L, Bousquet J. A pediatric asthma management program in a low-income setting resulting in reduced use of health service for acute asthma. *Allergy* 2010;65:1472-1477
16. Bunik M, Federico MJ, Beaty B, Rannie M, Olin JT, Kempe A. Quality improvement for asthma care within a hospital-based teaching clinic. *Academic Pediatrics* 2011;11:58-65
17. Swanson V, Wright S, Power KG, Duncan B, Morgan J, Turner E, *et al*. The impact of a structured programme of asthma care in general practice. *Int J Clin Pract* 2000;54:573-580
18. Findley SE, Thomas G, Madera-Reese R, McLeod N, Kintala S, Andres Martinez R, *et al.* A community-based strategy for improving asthma management and outcomes for preschoolers. *Journal of Urban Health* 2011;88 Suppl 1:S85-S99
19. Polivka BJ, Chaudry RV, Crawford J, Bouton P, Sweet L. Impact of an urban healthy homes intervention. *Journal of* *Environmental Health* 2011;73:16-20
