# Supplementary material for: Implementing supported self-management for asthma: a systematic review and suggested hierarchy of evidence of implementation studies
Source: BMC Med. 2015 Jun 1;13:127. doi: 10.1186/s12916-015-0361-0 (PMC4465463; doi:10.1186/s12916-015-0361-0)
Supplement: Additional file 5: — All results as reported in the included papers and the decision process underpinning the Harvest plot. [file 12916_2015_361_MOESM5_ESM.docx]

**Additional file 5.** **All results as reported in the included papers and the decision process underpinning the Harvest plot**

Where outcomes within a category were conflicting, the decision process attached priority as follows:

- Defined primary outcomes in an adequately powered study
- Outcomes that measured impact in the whole eligible population (typically using routine data rather than data from a sub-group who accepted/completed the intervention or were recruited for the evaluation)
- Outcomes which were measured with a validated instrument (as opposed to responses to non-validated questions)
- Outcomes that were clinically as well as statistically significant (e.g. achieved s defined minimum clinically important difference)

Finally, if there were any remaining doubt, the authors’ interpretation was considered as providing the context for our decision.

Abbreviations used in this table

Timepoints: I: Intervention vs C: Control B: Baseline vs FU: Follow-up

PAAP: Personalised asthma action plan

SABA: Short-acting beta_2_ agonists ICS: Inhaled corticosteroids

QoL: Quality of Life. miniAQLQ: mini Asthma Quality of Life Questionnaire PedsQL: Pediatric Quality of Life Inventory

ACQ: Asthma Control Questionnaire EQ5D: EuroQol 5D

ED: Emergency Department FEV_1_: Forced Expiratory Volume in one second

SD: standard deviation OR: Odds ratio HR: Hazard Ratio RR: Risk Ratio (or aRR: adjusted RR)

95%CI: 95% confidence interval NS: Not significant MCID: Minimum clinically important difference

| **Citation design, size and quality** | **Reported outcomes**  * indicates the primary outcome (if stated). | **Researcher’s interpretation for the Harvest plot** |
| --- | --- | --- |
| Cleland 2007[39]  Cluster RCT. FU: 6m  13 practices: 629 adults,  Quality score=24 | **Process outcomes**  Not assessed |  |
|  | **Asthma control**  * MiniAQLQ: (Mean and 95%CI) MCID is 0.5  Unadjusted. I: 6.41 (6.29 to 6.55) vs C: 6.31 (6.14 to 6.49) p=0.34  Adjusted for baseline and practice. I: 6.49 (6.40 to 6.59) vs C: 6.33 (6.23 to 6.44) p=0.03  [Authors comment that their ‘feasibility study was under-powered for a cluster RCT using miniAQLQ as a primary outcome’ ]  ACQ: (Mean and 95%CI) MCID is 0.5  Adjusted. I: 3.14 (3.05 to 3.23) vs C: 3.20 (3.10 to 3.30) p=0.43  SABA prescriptions (inhalers/patient)  Adjusted I: 5.57 (5.04 to 6.15) vs C: 5.77 (5.11 to 6.51) p=0.67  Oral steroid courses (courses/patient)  Adjusted I: 1.07 (1.04 to 1.10) vs C: 1.11 (1.07 to 1.45) p=0.12 | Adjusted mean difference in miniAQLQ (primary outcome; measured in a sub-group) was 0.16 which was statistically significant but less than the MCID of 0.5.  Other outcomes were negative.  [Authors describe their results as ‘disappointing’ and discuss the reasons why their intervention was ‘insufficient’ to effect change] **Illustrated as no effect,** **but hatched to indicate inconsistency** |
|  | **Unscheduled care**  Not assessed |  |
| Homer 2005 [30]  Cluster RCT. FU 12m  43 practices: 13,878 children  Quality score=18 | **Process outcomes**  * PAAP ownership (% with PAAP at baseline & FU)  At baseline I: 53% vs C: 37%. At follow-up I: 54% vs C: 41%  * Daily use of controller medications (% at baseline & FU)  At baseline I: 42% vs C: 38%. At follow-up I: 45% vs C: 39%  * Daily use of ICS (% at baseline & FU)  At baseline I: 17% vs C: 17%. At follow-up I: 15% vs C: 17% | 95% CI and/or between group significances were not reported for any outcomes. We therefore followed the conclusion of the authors that ‘After adjusting for state, practice size, child age, sex, and within-practice clustering, no overall effect of the intervention was found’  **Illustrated as consistently no effect in all three domains** |
|  | **Asthma control**  Parent reported exercise limitation (Scale 1 (limited) to 5)  Baseline I: 4.1 vs C: 4.2. Follow-up I: 4.2 vs C: 4.4  Parent reported asthma attacks (% with an attack in last year)  Baseline I: 57% vs C: 40%. Follow-up I: 54% vs C: 36% |  |
|  | **Unscheduled care**  Admissions (% with admission in last year)  At baseline I: 9% vs C: 2%. At follow-up I: 9% vs C: 4%  ED visits (% with ED visit in last year)  At baseline I: 36% vs C: 17%. At follow-up I: 36% vs C: 22% |  |
| Delaronde 2005 [32]  Preference RCT. FU 12  399 adults,  Quality score=20 | **Process outcomes**  Change in Asthma Medication Index (AMI) from routine data:  I: 0.18 vs C: 0.09 p=0.04  ‘Opt-in’: 0.29 (vs C p=0.01) ‘Opt-out’: 0.17, Non-respondents: 0.11 | Significant difference in change in AMI (derived from routine data on all eligible patients)  **Illustrated as a consistent significant positive effect** |
|  | **Asthma control**  Change in mean miniAQLQ: MCID is 0.5  I: 0.26 vs C: 0.12 ‘Opt-in’: 0.32, ‘Opt-out’: -0.08 | Authors state: ‘No significant between group differences’  **Illustrated as consistently no effect** |
|  | **Unscheduled care**  Admissions  Change in % with at least 1 admission: I: 1.5% vs C: 0%  ‘Opt-in’: 0% ‘Opt-out’: 0%, Non-respondents: 0%  ED attendances  Change in % with at least 1 ED attendance: I: 3.0% vs C: 3.0%  ‘Opt-in’: -10.6% ‘Opt-out’: 1.9%, Non-respondents: 2.7%  Physician office visits  Change in % with at least 1 admission: I: 11.9% vs C: -3.0%  ‘Opt-in’: 7.1% ‘Opt-out’: 13.4%, Non-respondents: -4.3% | Authors state ‘There were no statistically significant differences in the numbers of physician office visits, emergency department visits, or hospitalizations’  **Illustrated as consistently no effect** |
| Vollmer 2006 [35]  RCT,  6,948 adults,  Quality score=18 | **Process outcomes**  * ICS dispensed:  Proportion with ≥ 6 ICS canisters/year I: 30.4% vs C: 29.8% p=0.60  Self-reported ICS use at least several days a week:  I: 68.6% vs C: 70.7% p=0.20 | Neither objective ICS dispensing (primary outcome), nor self-reported ICS use showed significant between group difference  **Illustrated as consistently no effect** |
|  | **Asthma control**  * miniAQLQ: MCID is 0.5  I: 5.2 (SD 1.2) vs C: 5.1 (SD 1.2) p=0.48  Asthma Therapy Assessment  Proportion with no problems I: 54% vs C: 51% p=0.56  Asthma Impact Score:  I: 46.8 (SD 9.7) vs C: 47.4 (SD 9.4) p=0.46  Rescue medication use  Proportion with ≥ 6 SABA canisters/year I: 28.8% vs C: 29.0% p=0.86  Other, non-validated, outcomes (self-assessment of asthma severity, health status, confidence in ability to control, night waking knowledge and satisfaction with asthma care) no significant difference | Neither QoL (primary outcome) nor any other measure of control showed a significant between group difference.  **Illustrated as consistently no effect** |
|  | **Unscheduled care**  * Admission or ED visit:  Proportion with an admission I: 4.1% vs C: 4.0% p=0.88  Unscheduled consultations:  Proportion with unscheduled consultation I: 10.9% vs C: 10.0% p=0.28 | There was no between group difference in admissions ED attendances (primary outcome) or other use of healthcare resources  **Illustrated as consistently no effect** |
| Bunting 2006 [31]  Repeated measures study, 8yrs of routine data  207 adults,  Quality score=17 | **Process outcomes**  Self-reported PAAP ownership:  Proportion of patients B: 63% vs FU: 99% p<0.0001 | Significant increase in PAAP ownership.  **Illustrated as consistently positive** |
|  | **Asthma control**  Asthma severity by NAEPP guideline classification  Proportion classified as moderate/severe B: 77% vs FU: 49% p<0.001  55% improved over the study, 37% had no change, 8% were worse  Lung function:  Mean FEV_1_ % predicted B: 81% vs FU: 90% p<0.01  70% improved over the study, 6% had no change, 24% were worse  Non-validated questions (perception of severity, symptom scores) all improved significantly  Work absenteeism  Work days lost: B: 2.5/patient/year vs FU 0.5/patient/year | Significant improvement in severity classification, lung function and reduced says lost from work.  **Illustrated as consistently positive** |
|  | **Unscheduled care**  Hospitalisation or ED attendances  Three baseline years (Event/100 patients/year): 21.3, 22.2, 22.3 vs five FU years : 5.4, 2.6, 1.9, 5.4, 0  Proportion hospitalised each year: B: 4.0% vs FU 1.9%  Proportion attending ED each year: B: 9.9% vs FU 1.3% | Sustained reduction in hospitalisations and ED attendances (derived from routine data on whole populations).  **Illustrated as consistently positive** |
| Forshee 1998 [33]  Before and after study over 24 weeks  201 adults/children,  Quality score=15 | **Process outcomes**  Awareness, knowledge and confidence (self-reported, non-validated questions):  Scores on 5 knowledge questions (patients/parents) improved at the FU (no composite score)  ICS use  Increase in proportion on controller medication: adults 13.4% (p<0.001) and children 10.9% (p<0.01)  Routine care  Increase in proportion of adults attending monthly routine attendances: B: 33% to 59% (p<0.001)  Increase in proportion of children attending monthly routine attendances: B: 28% to 39% (p<0.001) | Significant increase in knowledge, routine care and use of controller medication.  **Illustrated as consistently positive** |
|  | **Asthma control**  Asthma severity: derived from validated Asthma Quality Assessment System (AQAS)  Improved from baseline to FU in adults (p<0.001) and children (p<0.01)  Generic QoL: from validated Asthma Quality Assessment System  Improved from baseline to FU in adults (p<0.001) and children (p<0.01)  Asthma specific QoL: from validated Asthma Quality Assessment System  Improved from baseline to FU in adults (p<0.001) and children (p<0.001)  Absenteeism from work/school  Proportion with lost days from school/work reduced: B: 20.5% vs FU: 18.0%  Days lost from work B: 6.5 vs FU: 3.9 (p<0.05)  Days lost from school B: 1.2 vs FU: 0.7 (NS) | Significant improvement in severity classification, generic and asthma-related QoL (derived from the validated AQAS). Days lost from work reduced, but reduction in school absenteeism not significant.  **Illustrated as consistently positive** |
|  | **Unscheduled care**  Admissions, ED attendances and unscheduled consultations  Proportion with unscheduled care reduced in adults (p<0.001) and children (p<0.001) | Reduction in unscheduled care.  **Illustrated as consistently positive** |
| Gerald 2006 [34]  Cluster RCT,  54 schools, 736 children,  quality score=18 | **Process outcomes**  Asthma knowledge.  School grades 1-3 increase score. Mean 3 points (range 0.2-4.1) p<0.0001  School grade 4 increase score Mean 0.7 points (range -0.6-1.4) p<0.0001 | Increase in knowledge amongst staff and children in 17 of the 18 schools  **Illustrated as consistently positive** |
|  | **Asthma control**  * School absences. Mean (SD) days/child/year  I: 3.88 (SD 3.5) vs C: 3.21 (SD 3.2) NS  School grades achieved Mean (SD)  I: 79.2 (SD 9.4) vs C: 80.3 (SD 8.5) NS | There was no between group difference in school absences or academic performance  **Illustrated as consistently no effect** |
|  | **Unscheduled care**  Hospitalisations (mean admissions/child (SD))  I: 0.04 (SD 0.19) vs C: 0.02 (SD 0.14) NS  ED attendances (mean visits/child (SD))  I: 0.09 (SD 0.28) vs C: 0.10 (SD 0.31) NS | There was no between group difference in use of healthcare resources  **Illustrated as consistently no effect** |
| Chini 2011[47]  Before-and-after study,  2,765 children: 135 with asthma,  Quality score=15 | **Process outcomes**  Not assessed |  |
|  | **Asthma control**  QoL. (Pediatric Quality of Life Inventory 4.0):  Children: B: 2.2 (SD 0.79) vs FU: 3.5 (SD 0.73) p<0.001  Parents: B: 3.1 (SD 0.6) vs FU: 3.5 (SD 0.4) p=0.004  Lung function  FEV_1_ % predicted: B:104.4% vs FU 102.5% p>0.05  Non-validated symptom score (day/night symptoms, asthma treatment, activity limitation) B: 86.1 (SD 6.5) vs FU: 94.9 (SD 6.1) p<0.001 | Significant improvement in validated QoL and non-validated symptom score. FEV_1_ was normal at baseline so no room for improvement.  **Illustrated as consistently positive** |
|  | **Unscheduled care**  Not assessed |  |
| Kemple 2003 [40]  RCT,  545 adults,  quality score=20 | **Process outcomes**  * Asthma review: Compared to control OR of a review (95%CI)  I(blank PAAP): OR 1.92 (1.18 to 3.11); I(PAAP): OR 2.33 (1.37 to 3.93)  * Knowledge: Compared to control OR of self-reported understanding of self-management (95%CI)  I(blank PAAP): OR 1.28 (0.66 to 2.45); I(PAAP): OR 2.20 (1.13 to 4.30)  Self-management discussed. Compared to control, OR of discussion of self-management (95%CI)  I(blank PAAP): OR 0.86 (0.53 to 1.39); I(PAAP): OR 0.83 (0.52 to 1.31)  ICS prescriptions (In 5 categories: 0, 1-2, 3-4, 5-9, 10 prescriptions/year)  OR of changing categories: I(blank PAAP): OR 1.31 (0.92 to 1.87); I(PAAP): OR 1.54 (1.06 to 2.25)  Proportion with ≥5 prescriptions: I(PAAP): 22%, I(AAP): 36% C: 39% NS | Inclusion of a PAAP resulted in positive benefit to the two primary outcomes (asthma review and knowledge about plans) and regular prescriptions of ICS  **Illustrated as consistently positive effect** |
|  | **Asthma control**  * Royal College of Physicians (RCP) 3 questions: Proportion with no symptoms  OR of changing score: I(blank PAAP): OR 1.43 (0.80 to 2.56); I(PAAP): OR 1.46 (0.81 to 2.61)  Proportion with no symptoms: I(blank PAAP): 21% vs I(PAAP): (21%) vs C: (16%)  Bronchodilator prescriptions. (In 6 categories: 0, 1-2, 3-4, 5-9, 10-19 >20 prescriptions/year)  OR of changing categories: I(blank PAAP): OR 1.29 (0.90 to 1.84); I(PAAP): OR 1.54 (0.80 to 1.68)  % with ≥5 prescriptions: I(blank PAAP): 43%, I(PAAP): 43% C: 39%  Steroid courses (In 3 categories: 0, 1, >1 courses/year)  OR of changing categories: I(blank PAAP): OR 0.62 (0.36 to 1.08); I(PAAP): OR 0.86 (0.50 to 1.46)  % with at least 1 course: I(blank PAAP): 28%, I(PAAP): 13% C: 20%  Peak flow (Median % predicted)  I(PAAP): 79%, I(PAAP): 81% C: 81% NS | There was no between group difference in markers of control (including the primary outcome of RCP3 questions measured in a sub-group)  **Illustrated as consistently no effect** |
|  | **Unscheduled care**  Admissions  % with at least 1 admission: I(PAAP): 2%, I(blank AAP): 0%, C: 2% NS  Unscheduled appointments  % with at least 1 out-of-hours consultation: I(PAAP): 1%, I(blank AAP): 2% C: 3% NS | There was no between group difference in use of healthcare resources  **Illustrated as consistently no effect** |
| Pinnock 2007 [41]  Controlled implementation trial,  1,809 adults and children,  Quality score=21 | **Process outcomes**  * Reviewed: Proportion with routine review  I: 66.4% vs C 53.8% ) risk difference 12.6% (95%CI 7.2 to 17.9)  Enablement: Mean (SD)  I: 7.29 (4.26) vs C: 6.43 (4.30) mean difference –0.83 (–1.56 to –0.10)  Confidence managing asthma: Mean (SD)  I: 3.99 (0.84) vs C: 3.78 (0.89) mean difference –0.21 (–0.36 to –0.06) | Telephone review option resulted in positive benefit to the primary outcome (asthma review) and measures of enablement and confidence (measured in a sub-group)  **Illustrated as consistently positive effect** |
|  | **Asthma control**  ACQ: Mean (SD) MCID is 0.5  I: 1.20 (1.00) vs C: 1.33 (1.13) mean difference 0.12 (–0.06 to 0.31)  miniAQLQ Mean (SD) MCID is 0.5  I: 5.29 (1.21) vs C: 5.31 (1.24) mean difference 0.02 (–0.21 to 0.24) | There was no between group difference in asthma control or QoL (measured in a sub-group with validated instruments)  **Illustrated as consistently no effect** |
|  | **Unscheduled care**  Not assessed |  |
| Lindberg 2002 [48]  Cross-sectional survey,  8 practices: 347 adults + random sample of 20/practice for survey  Quality score=16 | **Process outcomes**  *Clinical records*  6 of 7 criteria for good record keeping were significantly more likely to be in intervention practice notes  *Sub-group (survey)*  Non-validated questions about care as perceived by patients:  PAAP ownership I: 66% vs C: 45% p<0.001  Knowledge about asthma I: 91% vs C: 81% (p<0.01)  Taught inhaler technique I: 98% vs C: 96% (NS)  Take daily asthma medication I: 95% vs C: 90% (NS)  Use of a peak flow meter I: 84% vs C: 50% (p<0.001)  Information about prevention I: 89% vs C: 75% (p<0.001)  Named doctor I: 92% vs C: 94% (NS)  Automatic check up appointments I: 94% vs C: 80% (p<0.001) | Improved record keeping for 6 out 7 criteria in intervention practices. No defined primary outcome. 5 out of 8 management questions (non-validated, assessed in a sub-group) favoured the intervention: the 3 NS results were all over 90% at baseline so no room for improvement.  **Illustrated as positive effect** |
|  | **Asthma control**  EQ5D: No data given: Authors state that the EQ-5D showed ‘no significant differences in quality of life between the groups. There were only small variations around the average value of 0.86, which is close to the average value for the normal population’  Non-validated morbidity questions: in the last week:  Night-time awakening I: 26% vs C: 42% p < 0.01  Limitation in physical activity I: 17% vs C: 28% p < 0.05  Use of B2 agonist I: 57% vs C: 67% (NS)  More than two asthma attacks in last 6 months (%) I: 6% vs C: 12% p < 0.05  Increased sick leave in the intervention group (no data given) | The validated health status questionnaire showed no effect. 3 of the 5 (non-validated) morbidity questions favoured the intervention, but sick leave was greater in the intervention group.  **Illustrated as no effect, but hatched to indicate inconsistency** |
|  | **Unscheduled care**  Admissions: Proportion with an admission  I: 2.2% vs C: 3.7% NS  Consultations (not clear what proportion were unscheduled)  Proportion of patients: I: 43% vs C: 56% p<0.05. Visits/patient I: 1.25 vs 1.38 (NS) | Admissions and number of visits/patient showed no effect  **Illustrated as consistently no effect** |
| Haahtela 2006 [45]  10 year ITS,  Population of Finland,  Quality score=10 | **Process outcomes**  Self-management: Guided self-management was used more often (36% v 46%)  ICS use: Proportion using regular ICS increased: 33% in 1987 vs 85% in 2004  Ratio ICS/BD: increased steadily between 1992 and 1998 (from 0.8 to 1.2) | Increased prescribing of ICS and provision of self-management.  **Illustrated as consistently positive effect** |
|  | **Asthma control**  Absenteeism: fewer work days lost 1994: 145,200 lost days vs 2003: 105,700 days  Disability: 76% fewer people received disability pension (1993: 7212 (9%) vs 2003: 1741 (1.5%).  Deaths: Between 1993 and 2003, number of deaths fell from 123/yr to 85/yr  Death rate (n/person with asthma) fell from 0.91/1000 in 1993 to 0.41/1000 in 2003. | Reduction in sick days, registered disability and deaths from routine national data.  **Illustrated as consistently positive effect** |
|  | **Unscheduled care**  Admissions: Absolute reduction of 54% between 1993 and 2003 (from 110,000 to 51,000).  Admissions/person with asthma fell: 1993: 271/100,000 patients vs 2003: 120/100,000 patients  ED attendances: Between 1995 and 2003, ED visits decreased by 24% (adults) and 61% (children) | Reduction on admissions and ED attendances from national routine data  **Illustrated as consistently positive effect** |
| Kauppi 2012 [46]  10 year ITS,  Population of Finland,  Quality score=10 | **Process outcomes**  Prevalence: Diagnosed asthma has continued to rise (from 6.8% to 9.4%) | Prevalence of asthma continues to rise (also timeline overlaps with the full report (Haahtela 2006))  **Illustrated as consistently positive effect** |
|  | **Asthma control**  No outcomes |  |
|  | **Unscheduled care**  Admissions: Admissions fell from 32,000 hospital days in 2000, to 15,000 hospital days in 2010 | Routine national data on admissions  **Illustrated as consistently positive effect** |
| Souza Machado 2012 [44]  Controlled implementation study over 9 years,  Population of Salvador and Recife (control city),  Quality score=11 | **Process outcomes**  ICS use: The intervention (ProAR) dispensed 220,889 ICS inhalers over final 3 years of the intervention. ‘Strong’ inverse correlation between hospitalisation rates and drug dispensation (-0.801; p,0.001) | **Illustrated as consistently positive effect** |
|  | **Asthma control**  In-patient deaths: In Salvador, in-hospital asthma mortality decreased from 23 deaths/yr in 2003 to 1 in 2006. In Recife, the in-hospital mortality rate increased from 5 to 6 deaths/yr over the same time period. | Routine city level data  **Illustrated as consistently positive effect** |
|  | **Unscheduled care**  * Admissions  Hospitalisation rates/10,000 inhabitants: The rates of hospitalisation due to asthma at 9 years (2006) were 2.25 per 10,000 inhabitants in Salvador and 17.06 in Recife.  Hospitalisation rates declined by 82.3% in Salvador (from 1998: 12.72 in 1998 to 2.25 in 2006) vs 44.82% in the Recife (R250.906; p<0.001). After the ProAR intervention in 2003-2006 the rate of fall was -74.2% in Salvador vs -22.2% in Recife (p<0.001).  Admissions in children/adults: 4 yrs pre-ProAR intervention: admission rate fell 31.8% (children) and 38.6% (adults) vs 3yrs post-ProAR intervention: admission rate fell 68.2% (children) and 87.5% (adults) p=0.001 | Hospitalisation (primary outcome) obtained from national statistics  **Illustrated as consistently positive effect** |
| Andrade 2010 [43]  Before and after studyA,  582 children (470 cases and 112 controls)  Quality score=19 | **Process outcomes**  Use of ICS: Proportion of cases using ICS was 67% vs none of the control group  PAAPs: All the intervention users were given an action plan | Ownership of PAAPs and use of ICS increased from zero  **Illustrated as consistently positive effect** |
|  | **Asthma control**  No outcomes |  |
|  | **Unscheduled care**  * Unscheduled consultations:  Proportion with acute consultation: Cases: 5% vs Controls: 34% p<0.01.  Compared with controls, both ICS-users and non-ICS users of the intervention service were less likely to use unscheduled healthcare. HR for ICS-users 0.12 (95% CI, 0.07 to 0.21); HR for non-ICS users 0.04 (95% CI, 0.01–0.14) | Unscheduled appointments (primary outcome) were reduced in users of the intervention  **Illustrated as consistently positive effect** |
| Bunik 2011 [38]  5 year repeated measures study,  1,797 paediatric clinic attendees,  Quality score=15 | **Process outcomes**  * PAAP ownership: Proportion with a PAAP increased: 2006: 9% vs 2009: 42%  Compared to pre-intervention (2006), children seen in the 3 years after the intervention were more likely to receive a PAAP. 2007: aRR 2.29 (95%CI 2.03 to 2.56); 2008: aRR 2.40 (95% CI 2.15 to 2.66); 2009: aRR 2.86 (95% CI 2.60 to 3.20).  * Controller medication use: ‘Modest’ increase in proportion using controller medication: 2006: 61% vs 2009: 68%  Compared to pre-intervention (2006), children seen in the 3 years after the intervention were more likely to be prescribed controller medication. 2007: aRR 1.08 (95% CI 1.02 to 1.14; 2008: aRR 1.11 (95% CI 1.04 to 1.17); 2009: aRR 1.11 (95% CI 1.05 to 1.19).  * Record of severity: Proportion with severity recorded: 2006: 33% vs 2009: 74%  Compared to pre-intervention (2006), children seen in the 3 years after the intervention (2006) were more likely to have an assessment of asthma severity recorded 2007: aRR 1.31 (95%CI 1.26- to 1.36); 2008 aRR 1.44 (95% CI 1.38 to 1.50); 2009: aRR 1.47 (95% 1.41 to 1.54). | PAP ownership, use of controller medication and assessment of severity (primary outcomes) were all improved in the 4 years post-intervention.  **Illustrated as consistently positive effect** |
|  | **Asthma control**  No outcomes |  |
|  | **Unscheduled care**  Hospitalisation: No effect on proportion with an admission: 2006: 3% vs 2009: 3%  ED attendances: No effect on proportion with an ED attendances: 2006: 6% vs 2009: 6% | Proportion of children with an admission/ED attendance showed no effect  **Illustrated as consistently no effect** |
| Swanson 2000 [42]  Retrospective comparator study,  400 patients,  Quality score=16 | **Process outcomes**  PAAP ownership (patient reported):  Patients in intervention practices were more likely to have a PAAP I: 156 (63.7%) vs C: 71 (49.3%) (p<0.01)  Patients in intervention practices were more likely to have a peak flow meter I: 249 (87.1%) vs C: 119 (70.4%) (p<0.001)  Patients in intervention practices were more likely to follow an action plan all, most or some of the time: I: 160/303 vs C: 72/179 (p<0.01)  Routine care (from case notes)  Patients in intervention practices were more likely to have attended a routine review (I: mean attendances/patient/year 1.05 (SD1.6) vs C: 0.32 (SD0.32) p<0.05) | PAAP ownership, and regular review were all improved in the intervention practices.  **Illustrated as consistently positive effect** |
|  | **Asthma control**  Asthma symptoms (Patient reported: non-validated questions):  Attendees at intervention practice clinics reported fewer asthma symptoms (severity and control, sleep disturbance and early morning wheeze)(p<0.001) | **Illustrated as consistently positive effect** |
|  | **Unscheduled care**  Admissions (from case notes)  There was no significant change in the rate of admissions in either group. Intervention group: B: 0.06 (SD 0.4) vs FU: 0.03 (0.2) NS compared to no change in the control practices B: 0.04 (SD 0.3) vs FU: 0.03 (0.2) NS  ED attendances (from case notes)  There were fewer ED attendances/patient/year in patients from intervention practices at follow-up compared to baseline B: 0.24 (SD 0.8) vs FU: 0.11 (0.3) p<0.05 compared to no change in the control practices B: 0.07 (SD 0.3) vs FU: 0.08 (0.4) NS  Unscheduled consultations (from case notes)  There were fewer unscheduled consultations/patient/year in patients from intervention practices at follow-up compared to baseline B: 2.18 (SD 02.0) vs FU: 1.77 (1.8) p<0.05. The fall was similar in control practices B: 2.06 (SD 1.7) vs FU: 1.61 (1.7) p<0.01 | Reduction in emergency attendances and unscheduled care assessed from the clinical record. No change in the number of admissions (but these were very infrequent in this primary care population  **Illustrated as consistently positive effect** |
| Findlay 2011 [37]  Before-&-after study  35 centres, 1,908 children and their families,  Quality score=17 | **Process outcomes**  Knowledge and confidence:  Day care centre staff knowledge increased 49% to 82%;  Parents’ knowledge increased 62 to 79%.  Parents’ confidence increased from 57% to 81% (P <0.001)  PAAP use: Proportion of families with a PAAP increased from 46% to 57% (p=0.014) | PAAP ownership and knowledge both improved at the end of the year of the intervention.  **Illustrated as consistently positive effect** |
|  | **Asthma control**  Non-validated symptom questions:  Proportion with daytime symptoms dropped from 78% to 42% (p<0.001)  Proportion with nighttime symptoms decreased from 81% to 49% (p<0.001)  Time off school:  Proportion with any daycare absences dropped from 56% to 38% (p<0.001) | Symptoms and days off school due to asthma improved  **Illustrated as consistently positive effect** |
|  | **Unscheduled care**  Admissions  Proportion with any hospitalisation dropped from 24% to 11%. (p=0.001) | Admissions reduced significantly  **Illustrated as consistently positive effect** |
| Polivka 2011 [38]  Before-&-and after study,  243 families,  Quality score=18 | **Process outcomes**  Knowledge (non-validated questions):  Knowledge of asthma triggers (max = 18) B: 14.9 (2.8) vs FU: 16.5 (1.5) p<0.001  General asthma knowledge (max = 13) B: 9.6 (1.4) vs FU: 10.4 (1.5) p<0.001  Asthma trigger management activities (max = 18) B: 7.9 (2.7) vs FU: 12.4 (2.6) p<0.001  Caregiver self-efficacy (3 non-validated questions) B: 1.8 (SD 0.6) vs FU: 2.7 (SD 0.6) p<0.001  PAAP ownership: B: 25 (44%) vs FU: 38 (67%) p=0.007 | PAAP ownership, knowledge and trigger avoidance actions all improved significantly.  **Illustrated as consistently positive effect** |
|  | **Asthma control**  Non-validated symptom questions over previous 2 weeks:  Daytime symptoms in the previous 2 weeks: B: 5.0 (SD 4.2) vs FU 2.2 (SD 2.9) p<0.001  Nighttime symptoms in the previous 2 weeks: B: 3.6 (SD 4.1) vs FU: 1.8 (SD 3.1) p<0.001  Activity limited days in the previous 2 weeks: B: 4.1 (SD 4.6) vs FU: 1.7 (SD 3.0) p<0001  Child’s health (1 = excellent; 5 = poor): B: 3.0 (SD 1.0) vs FU: 2.4 (SD 1.0) p<0.001  School absenteeism in the previous 6 months: B: 5.3 (SD 9.2) vs FU: 1.4 (SD 2.7) p<0.001  Parental work absenteeism in the previous 6 months: B: 5.9 (SD 16.8) vs FU: 1.1 (SD 2.4) p=0.024  Caregiver QoL (9 non-validated questions): B: 25.9 (SD 6.6) vs FU: 29.5 (SD 6.0) p<0.001 | Reported symptoms, school /work absenteeism and caregiver QoL all improved significantly.  **Illustrated as consistently positive effect** |
|  | **Unscheduled care**  Admissions in the previous 3 months: B: 0.3 (SD 1.3) vs FU: 0.09 (SD 0.5) p=0.229  ED attendances in the previous 3 months: B: 1.7 (SD 2.7) vs FU: 0.4 (SD 0.7) p<0.001  Unscheduled consultations in the previous 3 months: B: 1.9 (SD 2.5) vs FU: 0.5 (SD 1.1) p<0.001 | ED attendances and unscheduled consultation significantly reduced. No impact on admissions.  **Illustrated as positive, but hatched to indicate inconsistency** |
